# Social marketing intervention for social media-related adolescent risk behaviours in North India: A multi-city cluster RCT protocol

**DOI:** 10.1371/journal.pone.0354663

**Published:** 2026-08-07

**Authors:** Tanvi Kiran, Ashrajdeep Singh, Divya Sharma, Aseem Mehra, Lakshay Jaglan, Tamanna Kumari, Pulak Syal, Madhu Gupta, Kapil Goel, Arun Kumar Aggarwal

**Affiliations:** 1 Department of Community Medicine and School of Public Health, Post Graduate Institute of Medical Education and Research (PGIMER), Chandigarh, India; 2 Department of Psychiatry, Post Graduate Institute of Medical Education and Research (PGIMER), Chandigarh, India; 3 Medical College, Government Medical College & Hospital, Chandigarh, India; PLOS: Public Library of Science, UNITED KINGDOM OF GREAT BRITAIN AND NORTHERN IRELAND

## Abstract

**Purpose:**

A majority of adolescents between the ages of 13 and 17 use at least one social media platform for interactive engagement. This age group marks a crucial period of social development, given the high susceptibility of young minds to influence. Excessive use of social media is associated with severe public health concerns, such as alcohol abuse, interpersonal dysfunction, and sexual behaviour-related concerns. This protocol details the development of a school-based social marketing intervention (SMI) and implementation of RCT to evaluate the effectiveness of SMI to reduce excessive social media use and associated adverse effects.

**Methods:**

The study will employ an open-label, cluster-randomized controlled trial (RCT) design. The study will be conducted in senior secondary schools located in three cities in Northern India (Chandigarh, Panchkula, and Mohali). A total of 12 schools will be selected, comprising four schools from each study area. Within each city, schools (clusters) will be randomized into intervention and control arms. Based on the available literature on sample size guidelines for Randomized Controlled Trials (RCTs), the total sample size is estimated to be 1260 students for all three cities. The SMI will be delivered in three phases: a pre-intervention assessment phase, a three-month intervention phase guided by the COM-B model of behavioural change, and a post-intervention evaluation phase to measure outcomes in both groups.

**Discussion:**

The adolescent relationship with social media is complex, providing both chances for learning and risks of misinformation and unhealthy behaviours. This study protocol aims to outline our plan to evaluate whether a structured SMI can enhance awareness and reduce adverse behaviours related to alcohol use, sexual health, and interpersonal relationships among adolescents.

**Trial Registration:**

Clinical Trial Registry-India, CTRI/2024/11/077115, originally registered on 11/11/2024.

## Introduction

Social media is now a fundamental part of everyone’s daily lives, particularly among adolescents [1]. Nearly 95% of adolescents use at least one social networking site, with 45% reporting they are online ‘almost consistently’ [2]. An Indian study also shows that almost 90% of adolescents have either a moderate or high level of social media addiction [3]. This constant exposure to the digital sphere makes it a critical public health concern.

The unregulated content of social media has a huge impact on the impressionable minds of teenagers. Numerous studies have linked excessive social media use among adolescents to a range of adverse outcomes, including alcohol abuse, disrupted family relationships, and risky sexual behaviours [4–6]. Early alcohol use among teenagers is associated with serious consequences, such as self-harm, aggression, and academic failure, with global DALYs for adolescent alcohol use coming to 2.80 billion in 2023 [7]. Also, Child-family relationships are adversely affected due to the time spent on social media by adolescents. There may be family disconnectedness, conflicts, lack of direct open communication, absence of cohesion, etc [8]. Exposure to sexualized content also poses risks; approximately 74.2% of the adolescents reported high or very high exposure to sexual content on social media that influences their sexual behaviour. Studies have found a link between media exposure and early initiation of sexual activity, which can lead to unintended pregnancies and increased vulnerability to sexually transmitted infections (STIs) [8].

While substantial research has been conducted in high-income countries to understand the relationship between social media use and adolescent behaviour, there is a growing need to explore these patterns in low- and middle-income countries (LMICs) like India, with an explosion of digital devices in a few years and a lack of school-based digital literacy programs. This demands culturally relevant, evidence-based interventions that can be easily implemented in a school curriculum.

The present protocol aims to investigate how a social marketing intervention will influence alcohol use, risky sexual behaviours, and adverse child-family relationships among school-going adolescents in India. Social marketing intervention (SMI) will be designed to promote healthier behaviours and equip adolescents with media literacy skills, refusal self-efficacy, and digital resilience. By leveraging the same persuasive techniques used in advertising, it can serve as a powerful tool to encourage positive behavioural change. The Social Marketing Intervention (SMI) will be developed through the collective contribution of stakeholders and will be pre-tested for maximum impact. The multi-cluster randomized controlled trial (RCT) will be employed to accurately assess effectiveness. Additionally, an economic evaluation will estimate the cost-effectiveness of the intervention, providing evidence for its sustainability and scalability within the existing programs, like the Ayushman Bharat School Health and Wellness Programme (SHWP).

## Methods

### Study aim and design

The study will employ an open-label, cluster-randomized controlled trial (RCT) design to investigate and evaluate the effects of a social marketing intervention for adverse child-family relationships, alcohol use disorders, and sexual health issues attributable to social media usage among school-going adolescents.

**Fig 1:**
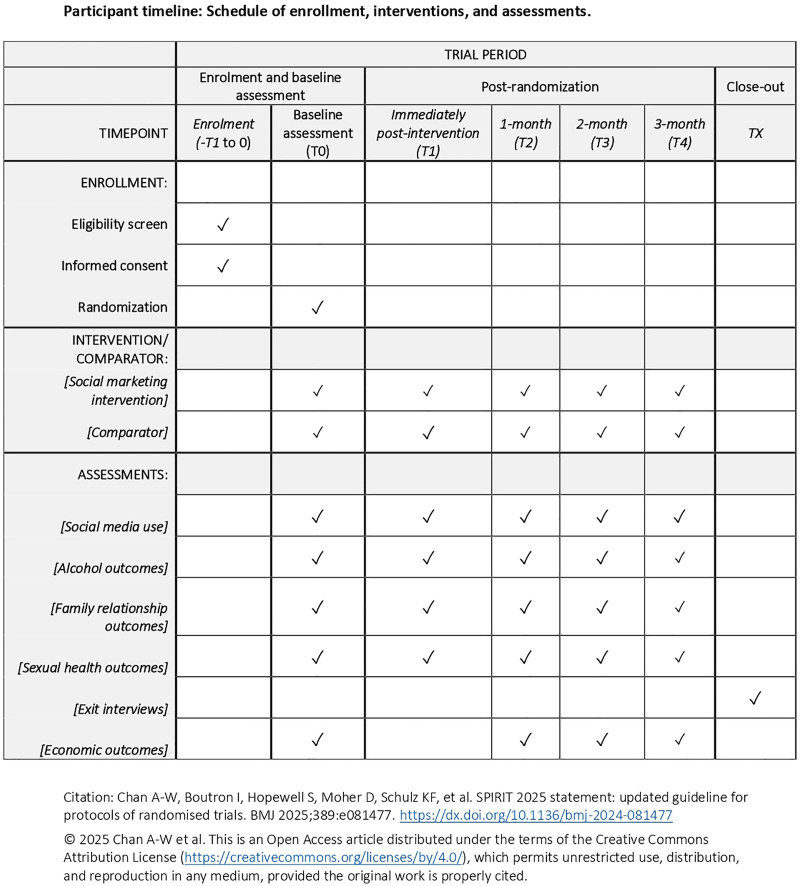
SPIRIT schedule and timeline.

A SPIRIT schedule of the study design can be found in Fig 1.

### Study area and settings

**Fig 2:**
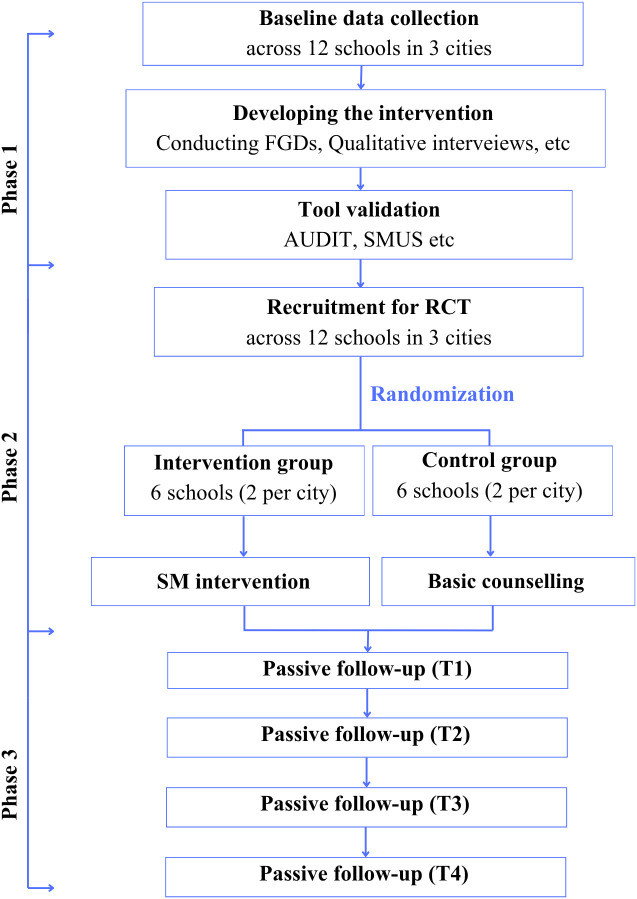
Implementation plan for the study.

The study will be conducted in senior secondary schools located in three cities in Northern India (Chandigarh, Panchkula, and Mohali). A total of 12 schools will be selected, comprising four schools from each study area. Within each city, schools (clusters) will be randomized into intervention and control arms (Fig 2). The protocol paper has been prepared following SPIRIT 2025 guidelines (S1 File).

### Participants

The study focuses on adolescents, specifically school students in the 9^th^, 10^th^, 11^th^, and 12^th^ grades (aged 14–17 years). The inclusion and exclusion criteria for the enrolment of study participants are described below:

#### Inclusion criteria.

- School-going adolescents aged 14–17 years, encompassing all genders.
- Adolescents who provide their assent along with parental consent to participate in this study.
- Adolescents who have access to smartphones/laptops/tablets and social media accounts.

#### Exclusion criteria.

- Students who do not have access to smartphones or internet access.
- An adolescent having been diagnosed with mental health problems or other chronic medical diseases.
- Adolescents who refuse to participate in the study.

### Trial registration and status

Clinical Trial Registry-India, CTRI/2024/11/077115, originally registered on 11/11/2024. The study has not yet generated results. Recruitment started on 01/11/2025 and will be completed tentatively by  31/12/2026. Data collection will be completed on 30/06/2027, and results are expected by 31/12/2027.

### Ethical approval and consent to participate

This study is part of a proposal approved by the Institutional Ethical Committee, Postgraduate Institute of Medical Education and Research (PGIMER), Chandigarh, India (IEC no. IEC-04/2024-3084, dated 14/10/2024). Prior to participation, written assent will be sought from all students, while written informed consent will be sought from their parents or legal guardians.

## Implementation plan

The social marketing intervention, implemented within the framework of a randomized controlled trial, will be carried out in three distinct phases (Fig 2):

1. Pre-intervention phase
2. Intervention phase
3. Post-intervention phase

### Phase 1: Pre-intervention phase

The baseline assessment will be done to estimate the time spent on social media, alcohol usage, adverse child-family relationships, and sexual behavior among adolescents.

#### Sample size for baseline assessment.

For Phase 1 (baseline assessment), the sample size is calculated using the formula n = 4pq/d^2^ [9]. Where n is the sample size for each area of Tricity, p is the prevalence, q is 1-p, and d is precision. The proportion of adolescents in India is approximately 20% [10]. The prevalence of alcohol consumption among adolescents at least once a year ranged from 10.6% to 32.9% [11]. *Prevalence of sexual activity ranged between 16.9%* [12] *to 21%, and sizable numbers of Indian adolescents were facing parent-child conflicts. Therefore,* assuming a prevalence of 20%, a precision of 4%, and an attrition rate of 5%, the target sample size is calculated as 420 for each of the three cities. Taking an equivalent number of samples from each of the three cities, the final sample size for all three study areas is 1260.

#### Sampling strategy for baseline assessment.

**Fig 3:**
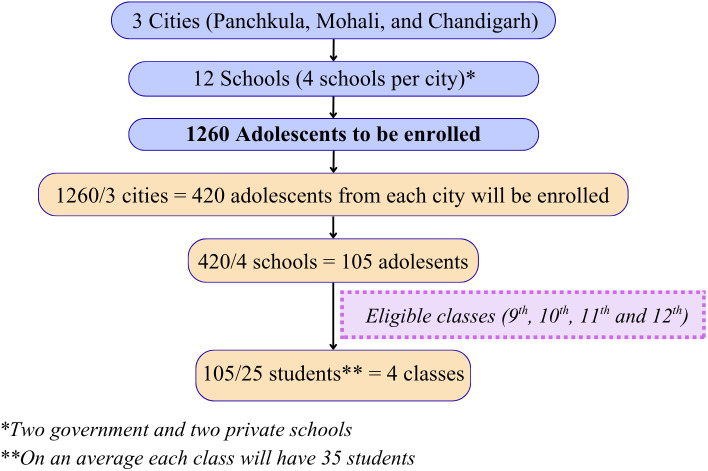
Sampling strategy of the study.

In total, 1260 adolescents will be recruited from 12 schools (4 schools per city) across three cities in Northern India (Fig 3). These four schools per city will be divided into two government schools and two private schools for socio-economic representation. Each city will recruit 420 adolescents, 105 students per school. We will randomly select 4 classes from the pool of eligible 9^th^-, 10^th^-, 11^th^, and 12th-grade classes, assuming an average class size ranging between 25 and 35 students.

#### Tools and materials used.

The assessment will be done by visiting the schools included in the sample and administering the standardized tools, including the Social Media Use Scale (SMUS) [13], Family Relationship Scales [8], Family Leisure Activity Profile [8], WHO’s Alcohol Use Disorders Identification Test (AUDIT) [14], and the Sexual Health Practice Tool. The questionnaire will undergo content validation in the North Indian context through expert review by professionals in public health, reproductive health, social sciences, and mental health. Pre-testing on 10% of the sample (≈120 adolescents) will ensure clarity, with language corrections as needed. Test-retest reliability will be measured using Cronbach’s Alpha. The details and early versions of the tools are attached in the S2 and S3 File.

#### Development and pre-testing of intervention.

The intervention will be developed and pre-tested using a mixed-methods approach, involving focus group discussions (FDGs), qualitative interviews, and pre-testing, with sample characteristics and sample size varying by stage.

According to Tynan and Drayton [14], 8–12 participants are needed for an FGD, with proportional selection of students, teachers, and parents. Following the Capability, Opportunity, Motivation-Behaviour (COM-B) model of behavioural change, the main aim of the FGD is to involve each stakeholder in assessing capability, opportunity, and motivation to change the behaviour of the school-going adolescents to address the issue of social media usage and its attributable adverse family-child relationship, alcohol usage, and sexual health issues. As per Boddy, a minimum of 6 FGDs in total will be conducted, with a minimum of 2 FGDs per study area, depending on the data saturation [15].

In total, 30 Qualitative interviews with the subject experts (mental health, social science & reproductive health) and the stakeholders of the intervention (students, parents & teachers) will be conducted [15]. Ten interviews per study area will be done with a proportionate number of stakeholders to identify the domains of the proposed social marketing intervention. For pretesting the intervention, a sample size of approximately 130 participants will be taken up (10% of the sample size for the intervention phase). The guides of the FGDs and qualitative interviews are attached in the S4 File.

#### Theoretical framework behind the intervention development.

This phase will follow an exploratory mixed-method study (qualitative formative research; quantitative content validity and pre-testing of the intervention) designed using the COM-B model of behavioural change. The COM-B system is a framework for understanding behaviour. In this ‘behaviour system,’ capability, opportunity, and motivation interact to generate behaviour that, in turn, influences these components [16]. Applying this to intervention design, the task is to identify the behavioural target and determine which components of the behaviour system need to be changed to achieve it.

#### Sensitization workshop.

Teachers will play a pivotal role as integral members in the course of implementing group sessions. A separate sensitization workshop will be conducted for teachers who will facilitate the intervention phase on behalf of the school. This workshop is designed to enable teachers to actively facilitate group sessions addressing crucial issues like excessive social media usage, child-family relationships, alcohol consumption, as well as sexual health issues, fostering a collaborative and engaging environment. By actively involving teachers in the execution of these sessions, the campaign becomes ingrained in the school's culture, ensuring its enduring impact.

### Phase 2: The Intervention Phase

#### Sample size for the intervention phase.

For Phase 2, based on the available literature on sample size guidelines for Randomized Controlled Trials. [17], for a 95% confidence level for the two-tailed hypothesis (${\text{Z}}_{\alpha }$= 1.96) and to achieve a power of 90% (${\text{Z}}_{\text{1}-\beta }$ = 1.2816), with an assumed standard deviation of population 0.15 and estimated effect size (D) of 5%, a minimum sample size of 189 per arm/group is required for the study, as per the following for the cluster-level randomization:


$$\begin{array}{c} \text{n}=\frac{{\;\text{2}\left({\text{Z}}_{\alpha }+{\text{Z}}_{\text{1}-\beta }\right)}^{\text{2}}\sigma^{\text{2}}}{(D{)}^{\text{2}}} \end{array}$$

By considering an attrition rate (10%), the sample size for each group (control and intervention) has come out to be approximately 208 participants in each of the control and intervention groups. Therefore, the minimum sample size for both control and intervention groups comes out to be 416 per study location. The total sample size is estimated to be n is equal to 1248, including all three cities in Northern India. To align with the baseline sample size, the RCT will also include 1,260 participants across all three cities.

#### Sampling strategy for randomized controlled trial.

The same COHORT from the pre-intervention phase (baseline assessment) will be followed. Therefore, the 12 schools will be randomly selected into 6 intervention groups and 6 control groups, as shown in Fig 2.

#### Open-label randomized control controlled trial.

Both the study participants and the researchers know which treatment is assigned to them. For the present study, blinding of the investigator and participants is not possible at the time of delivering the intervention, since the study aims to deliver a behaviour-centered intervention executed through a social marketing technique. Further, the investigator is required to build rapport with participants in the intervention group to deliver the intervention and motivate them to continue at each step. The participants shall also be aware that they will be given the intervention; therefore, the trial is open-label in nature. However, data shall be entered and analysed by a blinded/masked researcher.

#### Delivering the intervention.

A novel Social Marketing Intervention (SMI) will be provided for three months, once per month. The duration of each session will be between 1 hour and 1.5 hours. Social marketing is the application of marketing techniques and strategies to the analysis, planning, execution, and evaluation of programs designed to influence the voluntary behaviour of target audiences to improve their health outcomes, personal welfare, and that of their society [18]. The control group shall receive basic counselling consisting of awareness aimed at limiting social media use. An early version of the intervention is attached in the S5 File.

### Phase 3: Post-intervention phase

Assessments of outcomes will be conducted after completing the three-month intervention phase and for three months following the intervention closure. The Social Media Use Scale (SMUS), Family Relationship Scales, Family Leisure Activity Profile, WHO’s Alcohol Use Disorders Identification Test (AUDIT), and Sexual Health Practice Tool tools will be administered at baseline (T0) and at four time points after implementation of the intervention. The time points will include immediately post-intervention (T1) and at one-month post-intervention (T2), two months post-intervention (T3), and three months post-intervention (T4) for both the intervention and control groups. After three months of active intervention, the follow-up period will be passive. During the three-month follow-up, the intervention maintenance phase will be implemented. Additionally, the utilization of the Readiness to Behaviour Change Questionnaire is planned to assess the stage of behavioural change of students.

Exit interviews (approximately 50 interviews) will be conducted to elicit feedback and identify general challenges/barriers in executing the intervention from parents, teachers, and students as part of the research process

### Statistical analysis

All analyses will be conducted on a modified intention-to-treat (ITT) population, comprising all randomized participants with a completed baseline and at least one completed valid post-baseline assessment.

The study will incorporate the use of generalized estimating equations (GEE) as the regression model to analyze the changes in study outcomes (social media use and its attributable child-family relationship, alcohol consumption, as well as sexual health issues) over the specified periods. The formal mathematical framework and specification of the GEE model equations are provided in S6 File. This comprehensive approach will provide more accurate and reliable insights into the effectiveness of the intervention across different socio-demographic variables, economic variables, and time intervals in the data.

The GEE model shall be adjusted for different socio-demographic and economic variables as well. GEE is applicable for both continuous and categorical study outcomes, and in the present study, the outcomes are both metric and categorical. For categorical outcomes, the GEE procedure can be executed with the maximum likelihood estimation (MLE) algorithm described

Additionally, a repeated measures analysis of ANOVA shall be used to study the effectiveness of the intervention across different periods of intervention. The statistical analysis shall be carried out using Stata version 17. The results will be considered statistically significant if the P-value is less than 0.05.

Further, the qualitative assessment of the exit interviews to elicit feedback and identify general challenges/barriers in executing the intervention will be analysed using thematic analysis and transcript interpretation by employing open-source MAXQDA software.

### Economic evaluation

**Fig 4:**
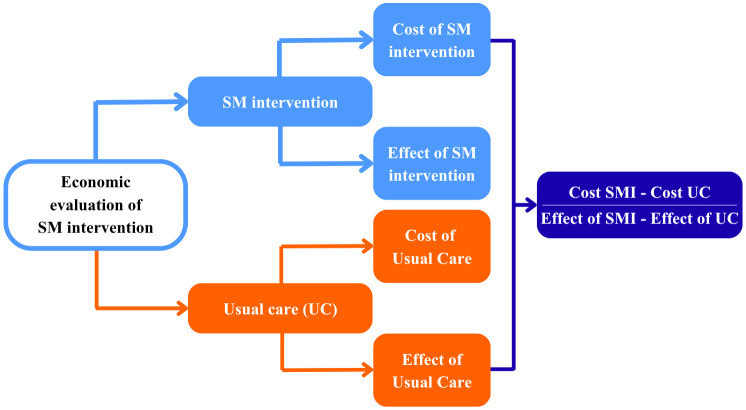
Economic evaluation of SM intervention.

For the economic evaluation of the developed intervention, both the costs and consequences (health outcomes) will be assessed using WHO’s CHOICE frameworks (Fig 4). The following costs and health outcomes shall be assessed:

- *Costs of the social marketing intervention*- The total cost in each scenario will be calculated from a societal perspective. The Intervention cost shall include programmatic costs (direct and indirect costs). The manpower costs shall be apportioned and annualized. It shall also include administrative costs, travel costs, quality assurance and monitoring, follow-up screening results, and logistics costs, etc. Thus, five-year costs for both intervention and comparator will be modelled using a hybrid economic evaluation simulation model.
- *Comparator*- The intervention will be compared to the hypothetical “null” scenario in which the effects of the currently implemented intervention will be removed.

A hybrid economic evaluation simulation model is proposed for this evaluation of the social marketing intervention developed under the study. The actual model will depend on the final study outcomes and parameters available. The data will be entered in Microsoft Excel 2019, and models will be built using TreeAge Pro.

The transition probabilities from the literature will be employed for the parameters within the model. Both costs and outcomes will be discounted at 3% annually, in line with international standards and prior Indian evaluations [19].

Cost-effectiveness analysis shall be undertaken. The endpoint for the CEA will be the Incremental Cost-Effectiveness Ratio. Cost-effectiveness is defined as the ratio of incremental costs to incremental outcomes. The incremental cost will be the difference between the total expected cost of the intervention and the total expected cost of no intervention [19]. The performance of interventions evaluated by this model will be measured using the cost-effectiveness ratio.

The following formula will be used for this computation:


$$\begin{array}{c} \text{ICER }=\;(\text{C2}-\text{C1})/(\text{E2}-\text{E1}) \end{array}$$

The numerator in the above equation implies the incremental costs, while the denominator implies the incremental effects. Here, C2 and C1 are the costs of intervention and comparator, respectively, while E2 and E1 are the effects of intervention and comparator, respectively. The incremental cost-effectiveness ratio per unit change in study outcomes will be calculated. A threshold between 1 and 3 times of GDP-Gross Domestic Product (as per WHO guidelines) may be used to consider the cost-effectiveness of the intervention, so as to consider for routine implementation as part of the national programme.

Both univariate and probabilistic sensitivity analysis will be conducted. Results from uncertainty analysis will be presented as a cost-effectiveness plane as well as a cost-effectiveness acceptability curve.

## Results chain of the study

**Fig 5:**
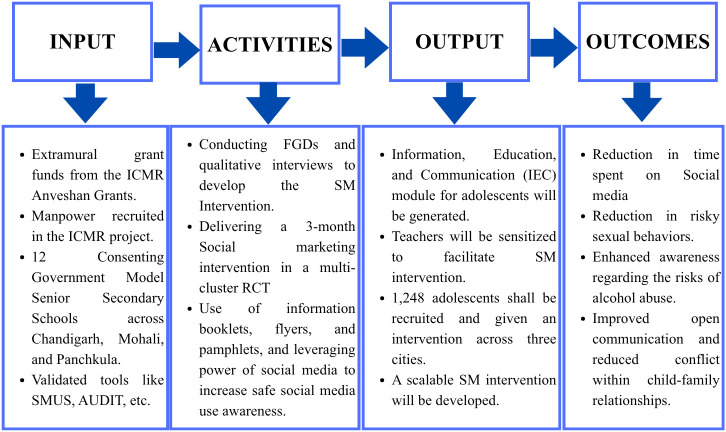
Result chain of the study.

This study protocol introduces a new approach to aware the adolescents about the harmful effects of excessive social media use. As shown in Fig 5, the inputs to the study will be extramural grant funds, staff for implementation of the study, schools, and validated tools. The program activities include designing the intervention, delivering the intervention, and assessing the change in behaviour at different intervals. The outputs will be newly developed IEC tools, teachers trained through sensitization workshops, and adolescents recruited in the intervention group. The outcomes consist of reduction in time spent on social media, reduction in risky sexual behaviour, enhanced awareness regarding the risks of alcohol use, and reduced conflict within child-family relationships.

## Potential problems and strategies

### Cultural sensitivity and addressing other challenges

Throughout the entire randomized controlled trial (RCT), we will prioritize cultural sensitivity, being particularly mindful of potential variations within different demographic groups. To ensure the robustness of our study results, we will implement separate sessions for both boys and girls, acknowledging and addressing any potential hesitation or discomfort that might arise in a mixed-gender setting. This approach aims to create an environment that respects the unique needs and perspectives of each gender group, allowing for more open and candid participation.

## Discussion

It is expected that the trial will generate a clearer understanding of how social media use is linked with alcohol-related behaviours, sexual health practices, and family dynamics in adolescents. In addition, it will create structured, evidence-based educational tools in the form of information, education, and communication (IEC) materials, which may be adapted for use in future school or community programs. A further anticipated outcome is the development of stronger self-regulation skills among participants, enabling them to make healthier and more deliberate choices in the digital space. Potential risks to participants are minimal, and strategies to manage these have been outlined in the “Potential Problems and Strategies” section.

In conclusion, targeted, well-designed interventions can help bridge the current gap in preventive efforts for young people. By combining interactive educational sessions with strategic use of social media marketing, this approach provides a practical and scalable method for promoting safer and more constructive online behaviour. Such measures have the potential to foster healthier lifestyles in adolescents, strengthen school and family support systems, and, over time, contribute to improved outcomes at both individual and societal levels.

## Supporting information

S1 File SPIRIT 2025 Checklist. (PDF)

S2 File Description of the study tools. (PDF)

S3 File Tentative version of the tools. (PDF)

S4 File FGDs and Interview guides. (PDF)

S5 File Tentative Components of the Proposed Social Marketing Intervention. (PDF)

S6 File GEE model equations. (PDF)
