# Supplementary material for: Social marketing intervention for social media-related adolescent risk behaviours in North India: A multi-city cluster RCT protocol
Source: PLoS One. 2026 Aug 7;21(8):e0354663. doi: 10.1371/journal.pone.0354663 (PMC13450755; doi:10.1371/journal.pone.0354663)
Supplement: Supplementary file 2 [file pone.0354663.s002.pdf]

Supplementary Material Appendix A: Tools used in the protocol

1. **Socio-demographic Data questionnaire-** To collect basic information of the participants, a sociodemographic data questionnaire, including questions on participants' details, including their parents' details, is included. The questionnaire also includes questions on social media access and use.
2. **Social Media Use Scale (SMUS)-** To estimate the time spent on social media by the participants, the Social Media Use Scale (SMUS) will be used. SMUS is a validated questionnaire consisting of 17 questions, which is scored on a Likert scale of 1-9, where 1=Never, 2=1-2 times per week, 3= 3-4 times per week, 4= 5-6 times per week, 5= once daily, 6= 2-5 times daily, 7= 6-9 times daily, 8=10-13 times daily, & 9= hourly or more. For scoring, there are four social media use subscales (Image-based, Comparison-based, Belief-based, and consumption-based). The scale shall be interviewer-administered. The items for each subscale should be averaged to get the final score. Spending More than 2 hours per day on social media is regarded as excessive use
3. **Family Systems and Family Open Communication scales** – The Family Systems scale is a self-administered, validated 9-item scale. Both the positive and negative impacts of social media on family systems and child-parental relationships were used to assess participants' perceptions of it. Respondents were asked to rate their agreement with each item on a 5-point Likert scale (1 = Strongly disagree; 5 = Strongly agree). The Open Communication scale, an 8-item, 5-point Likert scale, shall also be used to assess the adverse effects of social media on family communication. The scale shall be interviewer-administered.
4. **WHO's Alcohol Use Disorders Identification Test (AUDIT)** is a 10-item screening tool developed by the World Health Organization (WHO) to assess alcohol consumption, binge drinking behaviors (Consuming 5 or more drinks on an occasion for men or 4 or more drinks on an occasion for women), and alcohol dependence. A score of 8 or more is considered to indicate hazardous or harmful alcohol use (causing violence/injury under alcohol use). The AUDIT has been validated across genders and in a wide range of racial/ethnic groups and is well-suited for use in primary care settings. Further, the diagnostic criteria for alcohol abuse and dependence are detailed in the *Diagnostic and Statistical Manual of Mental Disorders of the American Psychiatric Association (DSM-IV)*. To meet the diagnosis of abuse, one of four abuse criteria must be met. To meet a diagnosis of dependence, three of seven dependence criteria must be met. The details of the criteria are mentioned explicitly in DSM (IV). The scale shall be interviewer-administered.
5. **The Sexual Health Practice Tool**- The Sexual Health Practice Tool is a novel, study-specific instrument developed by adapting relevant items from the SHAPE (Sexual Health and Adolescent Practice Evaluation) questionnaire. It comprises four sections: (A) Awareness/Knowledge – five items assessing adolescents' knowledge of how social media influences sexual health decisions; (B) Perception/Attitudes – five items assessing beliefs and attitudes toward sexual content on social media; (C) Sexual Health Practice/Behaviour – five items assessing behavioural exposure to sexual content; and (D) Relationships and Interpersonal Experiences – five items assessing how social media-

mediated romantic content shapes relationship perceptions. Sections A–C are scored on 5-point Likert scales. Section D includes a multiple-choice item on personal relationship experience. The tool is interviewer-administered and bilingual (English/Hindi).
