# Supplementary material for: Social marketing intervention for social media-related adolescent risk behaviours in North India: A multi-city cluster RCT protocol
Source: PLoS One. 2026 Aug 7;21(8):e0354663. doi: 10.1371/journal.pone.0354663 (PMC13450755; doi:10.1371/journal.pone.0354663)
Supplement: Supplementary file 3 [file pone.0354663.s003.pdf]

Social Marketing Intervention for adverse child-family relationships, Alcohol-usage and Sexual-Health issues attributable to Social-Media-Usage among Adolescents: A Multi-City Cluster Randomized Controlled Trial in North-India.

SOCIO-DEMOGRAPHIC DATA

S.NO.	STUDENT DETAILS	
1.	Name<br>नाम	
2.	Age<br>आयु	
3.	Gender<br>लिंग	• Male<br>पुरुष<br>• Female<br>महिला<br>• Other<br>अन्य
4.	Mobile No.<br>मोबाइल नंबर	
5.	Email ID<br>ईमेल आईडी	
6.	Place of residence<br>निवास की जगह	• Chandigarh<br>चंडीगढ़<br>• Mohali<br>मोहाली<br>• Panchkula<br>पंचकुला
7.	Class<br>कक्षा	• 9th<br>• 10th<br>• 11th<br>• 12th
8.	Class section/ Stream<br>कक्षा अनुभाग/ धारा	
9.	School name<br>विद्यालय का नाम	
10.	School recognized<br>विद्यालय मान्यता प्राप्त	• Government<br>सरकारी<br>• Govt. aided<br>सरकारी सहायता प्राप्त<br>• Private<br>प्राइवेट
	**FAMILY DETAILS**	
11.	Type of family<br>परिवार का प्रकार	• Nuclear family<br>एकल परिवार<br>• Joint family<br>संयुक्त परिवार<br>• Extended family<br>विस्तारित परिवार<br>• Living with a single parent<br>एकल माता-पिता के साथ रहना
12.	Number of Family Members<br>परिवार के सदस्यों की संख्या	
13.	Family income per month	

Funded By
Indian Council of Medical Research (ICMR)
(Ministry of Health and Family Welfare, Govt. of India)

Social Marketing Intervention for adverse child-family relationships, Alcohol-usage and Sexual-Health issues attributable to Social-Media-Usage among Adolescents: A Multi-City Cluster Randomized Controlled Trial in North-India.

SOCIAL MEDIA ACCESS AND USE			
14.	Do you engage in social media?<br>क्या आप सोशल मीडिया में सक्रिय हैं?	• Yes<br>हाँ<br>• No<br>नहीं	
15.	How much time do you spend on social media?<br>आप सोशल मीडिया पर कितना समय बिताते हैं?	• Daily (specify hours/minutes)<br>प्रतिदिन (घंटे/मिनट लिखें)	
		• Weekends/holidays (specify hours/minutes)<br>सप्ताहांत/अवकाश (घंटे/मिनट लिखें)	
16.	Social media use after 10 pm<br>रात 10 बजे के बाद सोशल मीडिया का उपयोग	• Never<br>कभी नहीं<br>• Occasionally<br>कभी-कभी<br>• Most nights<br>अधिकांश रातों में<br>• Every night<br>हर रात	
17.	Which social media platform do you use?<br>आप कौन-सा सोशल मीडिया प्लेटफ़ॉर्म उपयोग करते हैं?		
18.	Which of the following is used to engage in social media?<br>सोशल मीडिया उपयोग करने के लिए निम्न में से किसका उपयोग किया जाता है?	• Personal Smartphone<br>व्यक्तिगत स्मार्टफ़ोन<br>• Shared smartphone<br>साझा स्मार्टफ़ोन<br>• Tablet<br>टैबलेट<br>• Personal computer<br>व्यक्तिगत कंप्यूटर<br>• Shared computer<br>साझा कंप्यूटर	
19.	If you do not have your personal gadget, then whose gadget do you use?<br>यदि आपके पास अपना व्यक्तिगत गैजेट नहीं है, तो आप किसका गैजेट उपयोग करते हैं?	• Family (mother/father)<br>परिवार (माता/पिता)<br>• Grandparents<br>दादा-दादी / नाना-नानी<br>• Friend<br>मित्र<br>• Other, specify__________<br>अन्य, उल्लेख करें	
20.	Internet access at home<br>घर पर इंटरनेट की सुविधा	• Yes, unlimited<br>हाँ, असीमित<br>• Yes, limited<br>हाँ, सीमित<br>• Mobile-data only<br>केवल मोबाइल डेटा<br>• No internet<br>इंटरनेट नहीं	

Funded By
Indian Council of Medical Research (ICMR)
(Ministry of Health and Family Welfare, Govt. of India)

S.NO	FATHER/GUARDIAN DETAILS*		
**1.**	**Name**<br>नाम		
2.	Age<br>आयु		
3.	Mobile No.<br>मोबाइल नंबर		
4.	Email ID<br>ईमेल आईडी		
5.	Education<br>शिक्षा	• Illiterate<br>अशिक्षित<br>• Primary<br>प्राथमिक<br>• Middle<br>माध्यमिक<br>• High<br>उच्च<br>• Secondary/ diploma<br>माध्यमिक उत्तर/ उपाधि प्रमाणपत्र<br>• Graduate<br>स्नातक<br>• Post Graduation<br>स्नातकोत्तर<br>• Others:<br>अन्य _______________	
6.	Type of Occupation<br>रोज़गार का प्रकार	• Government _______________<br>सरकारी<br>• Private ____________________<br>प्राइवेट<br>• Self- Employed ____________<br>स्वयं-रोज़गार<br>• Others:<br>अन्य:_______________	
	**PARENTAL SOCIAL MEDIA USE**		
1.	Time spent on social media by parent<br>माता-पिता द्वारा सोशल मीडिया पर बिताया गया समय	• Daily (specify hours/minutes)<br>प्रतिदिन (घंटे/मिनट लिखें)	
		• Weekends/holidays (specify hours/minutes)<br>सप्ताहांत/अवकाश (घंटे/मिनट लिखें)	
2.	Parents' own use of social media after 10 pm<br>रात 10 बजे के बाद माता-पिता का स्वयं का सोशल मीडिया उपयोग	• Never<br>कभी नहीं<br>• Occasionally<br>कभी-कभी<br>• Most nights<br>अधिकांश रातों में<br>• Every night<br>हर रात	

Funded By
Indian Council of Medical Research (ICMR)
(Ministry of Health and Family Welfare, Govt. of India)

Social Marketing Intervention for adverse child-family relationships, Alcohol-usage and Sexual-Health issues attributable to Social-Media-Usage among Adolescents: A Multi-City Cluster Randomized Controlled Trial in North-India.

S.NO	MOTHER/GUARDIAN DETAIL*	
**1.**	**Name**<br>नाम	
2.	Age<br>आयु	
3.	Mobile No.<br>मोबाइल नंबर	
4.	Email ID<br>ईमेल आईडी	
5.	Education<br>शिक्षा	• Illiterate<br>अशिक्षित<br>• Primary<br>प्राथमिक<br>• Middle<br>माध्यमिक<br>• High<br>उच्च<br>• Secondary/ diploma<br>माध्यमिक उत्तर/ उपाधि प्रमाणपत्र<br>• Graduate<br>स्नातक<br>• Post Graduation<br>स्नातकोत्तर<br>• Others:<br>अन्य _______________
6.	Type of Occupation<br>रोज़गार का प्रकार	• Government _______________<br>सरकारी<br>• Private ____________________<br>प्राइवेट<br>• Self- Employed ____________<br>स्वयं-रोज़गार<br>• Others:<br>अन्य:_______________

PARENTAL SOCIAL MEDIA USE			
1.	Time spent on social media by parent<br>माता-पिता द्वारा सोशल मीडिया पर बिताया गया समय	• Daily (specify hours/minutes)<br>प्रतिदिन (घंटे/मिनट लिखें)	
		• Weekends/holidays (specify hours/minutes)<br>सप्ताहांत/अवकाश (घंटे/मिनट लिखें)	
2.	Parents' own use of social media after 10 pm<br>रात 10 बजे के बाद माता-पिता का स्वयं का सोशल मीडिया उपयोग	• Never<br>कभी नहीं<br>• Occasionally<br>कभी-कभी<br>• Most nights<br>अधिकांश रातों में<br>• Every night<br>हर रात	

Funded By
Indian Council of Medical Research (ICMR)
(Ministry of Health and Family Welfare, Govt. of India)

S. No	PARENTAL SOCIAL MEDIA USE SUPERVISION*	
1.	Parents are aware of the child's social media accounts<br>माता-पिता बच्चे के सोशल मीडिया अकाउंट्स से अवगत हैं	• Yes<br>हाँ<br>• No<br>नहीं
2.	Parents monitor the child's social media use<br>माता-पिता बच्चे के सोशल मीडिया उपयोग की निगरानी करते हैं	• Regularly check<br>नियमित रूप से जांचते हैं<br>• Occasionally check<br>कभी-कभी जांचते हैं<br>• Never check<br>कभी जांच नहीं करते
3.	Rules at home regarding phone/social media use<br>घर में फ़ोन/सोशल मीडिया उपयोग से संबंधित नियम	• Time limits<br>समय सीमाएँ<br>• No phone during meals<br>भोजन के समय फ़ोन नहीं<br>• No rules<br>कोई नियम नहीं<br>• Other___________<br>अन्य

***These sections will be cross-verified with the parents**

Funded By
Indian Council of Medical Research (ICMR)
(Ministry of Health and Family Welfare, Govt. of India)

SOCIAL MEDIA USE SCALE
(सोशल मीडिया उपयोग मापनी)

Directions: Please indicate how frequently you have engaged in each of the following social media activities in the **PAST WEEK** (7 days). Please only include activities engaged in on social networking sites such as Instagram, Facebook, etc.

S. No	Question	Never	1-2 times per week	3-4 times per week	5-6 times per week	Once daily	2-5 times daily	6-9 times daily	10-13 times daily	Hourly or more
1.	I posted or shared a story about something positive that was personally about me<br>मैंने अपने बारे में किसी सकारात्मक चीज़ पर पोस्ट या स्टोरी बनाई/साझा की									
2.	I looked at how many people liked, commented on, shared my content, or followed/friended me.<br>मैंने यह देखा कि कितने लोगों ने मेरे कंटेंट को पसंद किया, उस पर टिप्पणी की, साझा किया, या मुझे फॉलो/फ्रेंड किया									
3.	I read comments on my own content.<br>मैंने अपने स्वयं के कंटेंट पर की गई टिप्पणियाँ पढ़ीं									
4.	I edited or deleted my own social media content.<br>मैंने अपने सोशल मीडिया कंटेंट को संपादित किया या हटाया									
5.	I used photo filtering or photo editing<br>मैंने फोटो फ़िल्टरिंग या फोटो एडिटिंग का उपयोग किया									
6.	I compared my body or appearance to others'<br>मैंने अपने शरीर या रूप-रंग की तुलना दूसरों से की									
7.	I compared my life or experiences to others'<br>मैंने अपने जीवन या अनुभवों की तुलना दूसरों से की									
8.	I reminisced about the past<br>मैं अतीत की यादों में खोया									
9.	I posted or shared a story about something negative that was personally about me<br>मैंने अपने बारे में किसी नकारात्मक चीज़ पर पोस्ट या स्टोरी बनाई/साझा की									
10.	I posted or shared a story about something negative that was NOT personally about me									

Funded By
Indian Council of Medical Research (ICMR)
(Ministry of Health and Family Welfare, Govt. of India)

	मैंने अपने बारे में न होकर किसी अन्य नकारात्मक चीज़ पर पोस्ट या स्टोरी बनाई/साझा की									
11.	I commented unsupportively or disliked/reacted unsupportively to another person's post(s) मैंने दूसरों की पोस्ट पर असमर्थनात्मक टिप्पणी की या नापसंद/नकारात्मक प्रतिक्रिया दी									
12.	I sought out content that I morally or ethically disagreed with मैंने ऐसा कंटेंट ढूँढा जिससे मैं नैतिक या आचारिक रूप से असहमत था/थी									
13.	I scrolled aimlessly through my feed(s) मैंने बिना किसी उद्देश्य के अपने फ़ीड (feed) को स्क्रॉल किया									
14.	I looked at others' stories मैंने दूसरों की स्टोरीज़ देखीं									
15.	I navigated to others' profiles in my social network (e.g., friends or friends of friends) मैंने अपने सोशल नेटवर्क पर दूसरों की प्रोफ़ाइल देखीं (जैसे दोस्तों या दोस्तों के दोस्त)									
16.	I navigated to pages of people whom I do not know (e.g., influencers or other famous people) मैंने ऐसे लोगों के पेज देखे जिन्हें मैं नहीं जानता/जानती (जैसे इन्फ्लुएंसर या अन्य प्रसिद्ध लोग)									
17.	I watched videos such as memes, news content, tutorials/recipes, etc मैंने मीम्स, समाचार, ट्यूटोरियल/रेसिपी आदि जैसे वीडियो देखे									

SOCIAL MEDIA AND FAMILY RELATIONSHIPS SCALE

Scoring Instruction: 5-point Likert scale (1 = Strongly disagree; 5 = Strongly agree)
Please answer the following questions based on how your current social media use influences your relationships with your family.

S.No.	Question (सवाल)	Strongly Disagree (पूरी तरह असहमत)	Disagree (असहमत)	Neutral (निष्पक्ष)	Agree (सहमत)	Strongly Agree (पूरी तरह सहमत)
1.	Social media use has reduced face-to-face conversations among family members.<br>सोशल मीडिया के उपयोग ने परिवार के सदस्यों के बीच आमने-सामने की बातचीत को कम कर दिया है।					
2.	It often causes interruptions during shared family activities (e.g., meals, outings, or discussions).<br>यह अक्सर साझा पारिवारिक गतिविधियों (जैसे, भोजन, सैर या चर्चा) के दौरान रुकावटों का कारण बनता है।					
3.	It has made it harder for family members to engage in attentive listening.<br>इसने परिवार के सदस्यों के लिए ध्यान से सुनने में संलग्न होना कठिन बना दिया है।					
4.	The frequent use of social media has weakened emotional bonding and warmth within the family.<br>सोशल मीडिया के बार-बार इस्तेमाल ने परिवार के भीतर भावनात्मक बंधन और गर्मजोशी को कमजोर कर दिया है।					
5.	It has decreased family members' willingness to discuss personal concerns openly.<br>इसने परिवार के सदस्यों की व्यक्तिगत चिंताओं पर खुलकर चर्चा करने की इच्छा को कम कर दिया है।					
6.	Social media presence has limited opportunities for families to spend quality time together without digital distractions.<br>सोशल मीडिया की उपस्थिति में परिवारों के लिए डिजिटल विकर्षणों के बिना एक साथ गुणवत्तापूर्ण समय बिताने के सीमित अवसर हैं।					

Funded By
Indian Council of Medical Research (ICMR)
(Ministry of Health and Family Welfare, Govt. of India)

7.	Social media exposure has led to misunderstandings or conflicts between family members.<br>सोशल मीडिया एक्सपोजर ने परिवार के सदस्यों के बीच गलतफहमी या संघर्ष को जन्म दिया है।					
8.	Social media activities have raised concerns about family privacy and online safety.<br>सोशल मीडिया गतिविधियों ने पारिवारिक गोपनीयता और ऑनलाइन सुरक्षा के बारे में चिंताएं बढ़ा दी हैं।					
9.	Social media content has influenced the opinions and behaviours of children more strongly than parental guidance.<br>सोशल मीडिया सामग्री ने माता-पिता के मार्गदर्शन की तुलना में बच्चों की राय और व्यवहार को अधिक दृढ़ता से प्रभावित किया है।					
10.	It has affected mutual trust and transparency within the family.<br>इसने परिवार के भीतर आपसी विश्वास और पारदर्शिता को प्रभावित किया है।					

Funded By
Indian Council of Medical Research (ICMR)
(Ministry of Health and Family Welfare, Govt. of India)

FAMILY LEISURE ACTIVITY PROFILE

1.	**Do you have meals, at home, with family members?**<br>**क्या आप घर पर, परिवार के सदस्यों के साथ भोजन करते हैं? क्या आप घर पर, परिवार के सदस्यों के साथ भोजन करते हैं?**	• Yes<br>• No			
	If YES, how often? For about how long per time? (Check only one)<br>यदि हाँ, तो कितनी बार? प्रति समय लगभग कितने समय के लिए? (केवल एक की जाँच करें)यदि हाँ, तो कितनी बार? प्रति समय लगभग कितने समय के लिए? (केवल एक की जाँच करें)	• **At least daily**	< 1 hour	1–2 hrs	2–3 hrs
		• **At least weekly**	3–4 hrs	4–5 hrs	5–6 hrs
		• **At least monthly**	6–7 hrs	7–8 hrs	8–9 hrs
		• **At least annually**	9–10 hrs	>10 hrs	>1 day
	How satisfied are you with your participation with family members in these activities? (Please circle one)<br>आप इन गतिविधियों में परिवार के सदस्यों के साथ अपनी भागीदारी से कितने संतुष्ट हैं? (कृपया एक सर्कल करें)	• Very Dissatisfied<br>• Dissatisfied<br>• Neutral<br>• Satisfied<br>• Very Satisfied			
2.	**Do you participate in home-based activities (for example watching TV/videos, listening to music, reading books, singing, etc.) with family members**?<br>क्या आप परिवार के सदस्यों के साथ घर-आधारित गतिविधियों (उदाहरण के लिए टीवी/वीडियो देखना, संगीत सुनना, किताबें पढ़ना, गाना आदि) में भाग लेते हैं?	• Yes<br>• No			
	If YES, how often? For about how long per time? (Check only one)<br>यदि हाँ, तो कितनी बार? प्रति समय लगभग कितने समय के लिए? (केवल एक की जाँच करें)यदि हाँ, तो कितनी बार? प्रति समय लगभग कितने समय के लिए? (केवल एक की जाँच करें)	• **At least daily**	< 1 hour	1–2 hrs	2–3 hrs
		• **At least weekly**	3–4 hrs	4–5 hrs	5–6 hrs
		• **At least monthly**	6–7 hrs	7–8 hrs	8–9 hrs
		• **At least annually**	9–10 hrs	>10 hrs	>1 day
	How satisfied are you with your participation with family members in these activities? (Please circle one)<br>आप इन गतिविधियों में परिवार के सदस्यों के साथ अपनी भागीदारी से कितने संतुष्ट हैं? (कृपया एक सर्कल करें)	• Very Dissatisfied<br>• Dissatisfied<br>• Neutral<br>• Satisfied<br>• Very Satisfied			
3.	**Do you participate in games (for example playing cards, board games, video games, darts, billiards, etc.) with family members**?<br>क्या आप परिवार के सदस्यों के साथ खेलों में भाग लेते हैं (उदाहरण के लिए कार्ड, बोर्ड गेम, वीडियो गेम, डार्ट्स,	• Yes<br>• No			

Funded By
Indian Council of Medical Research (ICMR)
(Ministry of Health and Family Welfare, Govt. of India)

	बिलियर्ड्स, आदि) खेलना?				
	If YES, how often? For about how long per time? (Check only one)<br>यदि हाँ, तो कितनी बार? प्रति समय लगभग कितने समय के लिए? (केवल एक की जाँच करें)यदि हाँ, तो कितनी बार? प्रति समय लगभग कितने समय के लिए? (केवल एक की जाँच करें)	• **At least daily**	< 1 hour	1–2 hrs	2–3 hrs
		• **At least weekly**	3–4 hrs	4–5 hrs	5–6 hrs
		• **At least monthly**	6–7 hrs	7–8 hrs	8–9 hrs
		• **At least annually**	9–10 hrs	>10 hrs	>1 day
	How satisfied are you with your participation with family members in these activities? (Please circle one)<br>आप इन गतिविधियों में परिवार के सदस्यों के साथ अपनी भागीदारी से कितने संतुष्ट हैं? (कृपया एक सर्कल करें)	• Very Dissatisfied<br>• Dissatisfied<br>• Neutral<br>• Satisfied<br>• Very Satisfied			
4.	**Do you participate in crafts, cooking, and/or hobbies (for example drawing, scrap books, baking cookies, sewing, painting, ceramics, etc.) with family members?**<br>**क्या आप परिवार के सदस्यों के साथ शिल्प, खाना पकाने, और/या शौक (उदाहरण के लिए ड्राइंग, स्क्रैप किताबें, कुकीज़, बेकिंग, सिलाई, पेंटिंग, चीनी मिट्टी की चीज़ें, आदि) में भाग लेते हैं?**	• Yes<br>• No			
	If YES, how often? For about how long per time? (Check only one)<br>यदि हाँ, तो कितनी बार? प्रति समय लगभग कितने समय के लिए? (केवल एक की जाँच करें)यदि हाँ, तो कितनी बार? प्रति समय लगभग कितने समय के लिए? (केवल एक की जाँच करें)	• **At least daily**	< 1 hour	1–2 hrs	2–3 hrs
		• **At least weekly**	3–4 hrs	4–5 hrs	5–6 hrs
		• **At least monthly**	6–7 hrs	7–8 hrs	8–9 hrs
		• **At least annually**	9–10 hrs	>10 hrs	>1 day
	How satisfied are you with your participation with family members in these activities? (Please circle one)<br>आप इन गतिविधियों में परिवार के सदस्यों के साथ अपनी भागीदारी से कितने संतुष्ट हैं? (कृपया एक सर्कल करें)	• Very Dissatisfied<br>• Dissatisfied<br>• Neutral<br>• Satisfied<br>• Very Satisfied			
5.	**Do you participate in music, dance, and drama (for example dancing, playing musical instruments, singing, acting, etc.) with family members?**<br>**क्या आप परिवार के सदस्यों के साथ संगीत, नृत्य और नाटक (उदाहरण के लिए नृत्य, संगीत वाद्ययंत्र बजाना, गायन, अभिनय आदि) में भाग लेते हैं?**	• Yes<br>• No			

<table>
<tr><td rowspan="4"></td><td rowspan="4">If YES, how often? For about how long per time? (Check only one)
यदि हाँ, तो कितनी बार? प्रति समय लगभग कितने समय के लिए? (केवल एक की जाँच करें)यदि हाँ, तो कितनी बार? प्रति समय लगभग कितने समय के लिए? (केवल एक की जाँच करें)</td><td>• At least daily</td><td>< 1 hour</td><td>1–2 hrs</td><td>2–3 hrs</td></tr>
<tr><td>• At least weekly</td><td>3–4 hrs</td><td>4–5 hrs</td><td>5–6 hrs</td></tr>
<tr><td>• At least monthly</td><td>6–7 hrs</td><td>7–8 hrs</td><td>8–9 hrs</td></tr>
<tr><td>• At least annually</td><td>9–10 hrs</td><td>>10 hrs</td><td>>1 day</td></tr>
<tr><td></td><td>How satisfied are you with your participation with family members in these activities? (Please circle one)
आप इन गतिविधियों में परिवार के सदस्यों के साथ अपनी भागीदारी से कितने संतुष्ट हैं? (कृपया एक सर्कल करें)</td><td colspan="4">• Very Dissatisfied
• Dissatisfied
• Neutral
• Satisfied
• Very Satisfied</td></tr>
<tr><td>6.</td><td>Do you participate in sports and outdoor activities (for example swimming, skating, jogging, biking, hiking, skiing, etc.) with family members?
क्या आप परिवार के सदस्यों के साथ खेल और बाहरी गतिविधियों (उदाहरण के लिए तैराकी, स्केटिंग, जॉगिंग, बाइकिंग, लंबी पैदल यात्रा, स्कीइंग, आदि) में भाग लेते हैं?</td><td colspan="4">• Yes
• No</td></tr>
<tr><td rowspan="4"></td><td rowspan="4">If YES, how often? For about how long per time? (Check only one)
यदि हाँ, तो कितनी बार? प्रति समय लगभग कितने समय के लिए? (केवल एक की जाँच करें)यदि हाँ, तो कितनी बार? प्रति समय लगभग कितने समय के लिए? (केवल एक की जाँच करें)</td><td>• At least daily</td><td>< 1 hour</td><td>1–2 hrs</td><td>2–3 hrs</td></tr>
<tr><td>• At least weekly</td><td>3–4 hrs</td><td>4–5 hrs</td><td>5–6 hrs</td></tr>
<tr><td>• At least monthly</td><td>6–7 hrs</td><td>7–8 hrs</td><td>8–9 hrs</td></tr>
<tr><td>• At least annually</td><td>9–10 hrs</td><td>>10 hrs</td><td>>1 day</td></tr>
<tr><td></td><td>How satisfied are you with your participation with family members in these activities? (Please circle one)
आप इन गतिविधियों में परिवार के सदस्यों के साथ अपनी भागीदारी से कितने संतुष्ट हैं? (कृपया एक सर्कल करें)</td><td colspan="4">• Very Dissatisfied
• Dissatisfied
• Neutral
• Satisfied
• Very Satisfied</td></tr>
<tr><td>7.</td><td>Do you participate in trips and excursions (for example outings, picnics, movies, eating out, camping, traveling, etc.) with family members?
क्या आप परिवार के सदस्यों के साथ यात्राओं और भ्रमण (उदाहरण के लिए सैर, पिकनिक, फिल्में, बाहर खाना, शिविर, यात्रा आदि) में भाग लेते हैं?</td><td colspan="4">• Yes
• No</td></tr>
</table>

Funded By
Indian Council of Medical Research (ICMR)
(Ministry of Health and Family Welfare, Govt. of India)

	If YES, how often? For about how long per time? (Check only one)<br>यदि हाँ, तो कितनी बार? प्रति समय लगभग कितने समय के लिए? (केवल एक की जाँच करें)यदि हाँ, तो कितनी बार? प्रति समय लगभग कितने समय के लिए? (केवल एक की जाँच करें)	• **At least daily**	< 1 hour	1–2 hrs	2–3 hrs
		• **At least weekly**	3–4 hrs	4–5 hrs	5–6 hrs
		• **At least monthly**	6–7 hrs	7–8 hrs	8–9 hrs
		• **At least annually**	9–10 hrs	>10 hrs	>1 day
	How satisfied are you with your participation with family members in these activities? (Please circle one)<br>आप इन गतिविधियों में परिवार के सदस्यों के साथ अपनी भागीदारी से कितने संतुष्ट हैं? (कृपया एक सर्कल करें)	• Very Dissatisfied<br>• Dissatisfied<br>• Neutral<br>• Satisfied<br>• Very Satisfied			
8.	**Do you participate in community and religious activities (for example church/mosque/temple events, volunteering, social gatherings, etc.) with family members?**<br>**क्या आप परिवार के सदस्यों के साथ सामुदायिक और धार्मिक गतिविधियों (उदाहरण के लिए चर्च/मस्जिद/मंदिर कार्यक्रमों, स्वयंसेवा, सामाजिक समारोहों आदि) में भाग लेते हैं?**	• Yes<br>• No			
	If YES, how often? For about how long per time? (Check only one)<br>यदि हाँ, तो कितनी बार? प्रति समय लगभग कितने समय के लिए? (केवल एक की जाँच करें)यदि हाँ, तो कितनी बार? प्रति समय लगभग कितने समय के लिए? (केवल एक की जाँच करें)	• **At least daily**	< 1 hour	1–2 hrs	2–3 hrs
		• **At least weekly**	3–4 hrs	4–5 hrs	5–6 hrs
		• **At least monthly**	6–7 hrs	7–8 hrs	8–9 hrs
		• **At least annually**	9–10 hrs	>10 hrs	>1 day
	How satisfied are you with your participation with family members in these activities? (Please circle one)<br>आप इन गतिविधियों में परिवार के सदस्यों के साथ अपनी भागीदारी से कितने संतुष्ट हैं? (कृपया एक सर्कल करें)	• Very Dissatisfied<br>• Dissatisfied<br>• Neutral<br>• Satisfied<br>• Very Satisfied			

9.	**Do you celebrate festivals, birthdays, or special occasions with family members?**<br>**क्या आप परिवार के सदस्यों के साथ त्योहार, जन्मदिन या विशेष अवसर मनाते हैं?**	• Yes<br>• No			
	If YES, how often? For about how long per time? (Check only one)<br>यदि हाँ, तो कितनी बार? प्रति समय	• **At least daily**	< 1 hour	1–2 hrs	2–3 hrs
		• **At least weekly**	3–4 hrs	4–5 hrs	5–6 hrs
		• **At least monthly**	6–7 hrs	7–8 hrs	8–9 hrs

Funded By
Indian Council of Medical Research (ICMR)
(Ministry of Health and Family Welfare, Govt. of India)

	लगभग कितने समय के लिए? (केवल एक की जाँच करें)यदि हाँ, तो कितनी बार? प्रति समय लगभग कितने समय के लिए? (केवल एक की जाँच करें)	• **At least annually**	9–10 hrs	>10 hrs	>1 day
	How satisfied are you with your participation with family members in these activities? (Please circle one)<br>आप इन गतिविधियों में परिवार के सदस्यों के साथ अपनी भागीदारी से कितने संतुष्ट हैं? (कृपया एक सर्कल करें)	• Very Dissatisfied<br>• Dissatisfied<br>• Neutral<br>• Satisfied<br>• Very Satisfied			
10.	**Do you go shopping (for clothes, groceries, household items, etc.) with family members?**<br>**क्या आप परिवार के सदस्यों के साथ खरीदारी (कपड़े, किराने का सामान, घरेलू सामान आदि के लिए) जाते हैं?**	• Yes<br>• No			
	If YES, how often? For about how long per time? (Check only one)<br>यदि हाँ, तो कितनी बार? प्रति समय लगभग कितने समय के लिए? (केवल एक की जाँच करें)यदि हाँ, तो कितनी बार? प्रति समय लगभग कितने समय के लिए? (केवल एक की जाँच करें)	• **At least daily**	< 1 hour	1–2 hrs	2–3 hrs
		• **At least weekly**	3–4 hrs	4–5 hrs	5–6 hrs
		• **At least monthly**	6–7 hrs	7–8 hrs	8–9 hrs
		• **At least annually**	9–10 hrs	>10 hrs	>1 day
	How satisfied are you with your participation with family members in these activities? (Please circle one)<br>आप इन गतिविधियों में परिवार के सदस्यों के साथ अपनी भागीदारी से कितने संतुष्ट हैं? (कृपया एक सर्कल करें)	• Very Dissatisfied<br>• Dissatisfied<br>• Neutral<br>• Satisfied<br>• Very Satisfied			
11.	**Do you help family members in daily chores or responsibilities (for example cleaning, cooking, gardening, caring for younger/older family members)?**<br>**क्या आप परिवार के सदस्यों को दैनिक कामों या जिम्मेदारियों में मदद करते हैं (उदाहरण के लिए सफाई, खाना पकाना, बागवानी, परिवार के छोटे/बड़े सदस्यों की देखभाल करना)?**	• Yes<br>• No			
	If YES, how often? For about how long per time? (Check only one)<br>यदि हाँ, तो कितनी बार? प्रति समय लगभग कितने समय के लिए? (केवल एक की जाँच करें)यदि हाँ, तो कितनी बार? प्रति समय लगभग कितने समय के लिए? (केवल एक की जाँच करें)	• **At least daily**	< 1 hour	1–2 hrs	2–3 hrs
		• **At least weekly**	3–4 hrs	4–5 hrs	5–6 hrs
		• **At least monthly**	6–7 hrs	7–8 hrs	8–9 hrs
		• **At least annually**	9–10 hrs	>10 hrs	>1 day

Funded By
Indian Council of Medical Research (ICMR)
(Ministry of Health and Family Welfare, Govt. of India)

	How satisfied are you with your participation with family members in these activities? (Please circle one)<br>आप इन गतिविधियों में परिवार के सदस्यों के साथ अपनी भागीदारी से कितने संतुष्ट हैं? (कृपया एक सर्कल करें)	• Very Dissatisfied<br>• Dissatisfied<br>• Neutral<br>• Satisfied<br>• Very Satisfied			
12.	**Do you participate in family discussions (for example decision making, planning, problem-solving, etc.) with family members?**<br>**क्या आप परिवार के सदस्यों के साथ पारिवारिक चर्चाओं (उदाहरण के लिए निर्णय लेने, योजना, समस्या-समाधान, आदि) में भाग लेते हैं?**	• Yes<br>• No			
	If YES, how often? For about how long per time? (Check only one)<br>यदि हाँ, तो कितनी बार? प्रति समय लगभग कितने समय के लिए? (केवल एक की जाँच करें)यदि हाँ, तो कितनी बार? प्रति समय लगभग कितने समय के लिए? (केवल एक की जाँच करें)	• **At least daily**	< 1 hour	1–2 hrs	2–3 hrs
		• **At least weekly**	3–4 hrs	4–5 hrs	5–6 hrs
		• **At least monthly**	6–7 hrs	7–8 hrs	8–9 hrs
		• **At least annually**	9–10 hrs	>10 hrs	>1 day
	How satisfied are you with your participation with family members in these activities? (Please circle one)<br>आप इन गतिविधियों में परिवार के सदस्यों के साथ अपनी भागीदारी से कितने संतुष्ट हैं? (कृपया एक सर्कल करें)	• Very Dissatisfied<br>• Dissatisfied<br>• Neutral<br>• Satisfied<br>• Very Satisfied			
13.	**Do you participate in any other family activities not listed above**?<br>क्या आप किसी अन्य पारिवारिक गतिविधियों में भाग लेते हैं जो ऊपर सूचीबद्ध नहीं हैं?	• Yes<br>• No			
	If YES, please specify:<br>यदि हाँ, तो कृपया निर्दिष्ट करें:				
	How satisfied are you with your participation with family members in these activities? (Please circle one)<br>आप इन गतिविधियों में परिवार के सदस्यों के साथ अपनी भागीदारी से कितने संतुष्ट हैं? (कृपया एक सर्कल करें)	• Very Dissatisfied<br>• Dissatisfied<br>• Neutral<br>• Satisfied<br>• Very Satisfied			

Funded By
Indian Council of Medical Research (ICMR)
(Ministry of Health and Family Welfare, Govt. of India)

Social Marketing Intervention for adverse child-family relationships, Alcohol-usage and Sexual-Health issues attributable to Social-Media-Usage among Adolescents: A Multi-City Cluster Randomized Controlled Trial in North-India.

WHO's Alcohol Use Disorders Identification Test -Modified (AUDIT SCALE)

Questions	0	1	2	3	4
1. How often do you have a drink containing alcohol, following exposure to alcohol-related content on social media?<br>सोशल मीडिया पर शराब से संबंधित सामग्री के संपर्क में आने के बाद आप कितनी बार शराब से युक्त पेय पीते हैं?	Never (skip to Q 9)	Monthly or less	2-4 times a month	2-3 times a week	4 or more times a week
2. On a typical day when you drink alcohol, how many drinks do you usually have when your drinking is influenced by social media content?<br>एक सामान्य दिन जब आप शराब पीते हैं, तो आप आमतौर पर कितने पेय पीते हैं जब आपका शराब सोशल मीडिया सामग्री से प्रभावित होता है?	1 or 2	3 or 4	5 or 6	7 to 9	10 or more
3. How often do you have six or more drinks on one occasion after feeling encouraged by alcohol-related content on social media?<br>सोशल मीडिया पर शराब से संबंधित सामग्री से प्रोत्साहित महसूस करने के बाद आप एक अवसर पर कितनी बार छह या अधिक पेय पीते हैं?	Never	Less than monthly	Monthly	Weekly	Daily or almost daily
4. How often during the last year have you found that you were not able to stop drinking once you started, in situations where social media content influenced your drinking?<br>पिछले वर्ष के दौरान आपने कितनी बार पाया है कि आप एक बार शुरू करने के बाद शराब पीना बंद करने में सक्षम नहीं थे, उन स्थितियों में जहां सोशल मीडिया सामग्री ने आपके पीने को प्रभावित किया?	Never	Less than monthly	Monthly	Weekly	Daily or almost daily
5. How often during the last year have you failed to do what was normally expected of you after drinking that was influenced by social media content?<br>पिछले वर्ष के दौरान कितनी बार आप वह करने में विफल रहे हैं जो पीने के बाद आपसे सामान्य रूप से अपेक्षित था जो सोशल मीडिया सामग्री से प्रभावित था?	Never	Less than monthly	Monthly	Weekly	Daily or almost daily
6. How often during the last year have you felt the need to have a first drink in the morning to get yourself going after a night of drinking influenced by social media content?<br>पिछले वर्ष के दौरान कितनी बार आपने सोशल मीडिया सामग्री से प्रभावित रात भर शराब पीने के बाद खुद को पाने के लिए सुबह पहला पेय लेने की आवश्यकता महसूस की है?	Never	Less than monthly	Monthly	Weekly	Daily or almost daily

Funded By
Indian Council of Medical Research (ICMR)
(Ministry of Health and Family Welfare, Govt. of India)

7. How often during the last year have you felt guilt or remorse about drinking that was influenced by alcohol-related content on social media?<br>पिछले वर्ष के दौरान आपने कितनी बार शराब पीने के बारे में अपराधबोध या पछतावा महसूस किया है जो सोशल मीडिया पर शराब से संबंधित सामग्री से प्रभावित था?	Never	Less than monthly	Monthly	Weekly	Daily or almost daily
8. How often during the last year have you been unable to remember what happened the night before after drinking influenced by social media content?<br>पिछले वर्ष के दौरान कितनी बार आप यह याद नहीं रख पाए हैं कि सोशल मीडिया सामग्री से प्रभावित शराब पीने के बाद एक रात पहले क्या हुआ था?	Never	Less than monthly	Monthly	Weekly	Daily or almost daily
9. Have you or someone else ever been injured following alcohol use that was influenced by social media content?<br>क्या आप या कोई और कभी शराब के उपयोग के बाद घायल हो गया है जो सोशल मीडिया सामग्री से प्रभावित था?	No		Yes but not in the last year		Yes, during the last year
10. Has a relative, friend, doctor, or health worker expressed concern about your drinking in relation to social media influence or suggested that you cut down?	No		Yes but not in the last year		Yes, during the last year

Binge drinking behaviors (Consuming 5 or more drinks on an occasion for men or 4 or more drinks on an occasion for women). Total scores of 8 or more are recommended as indicators of hazardous and harmful alcohol use.

Funded By
Indian Council of Medical Research (ICMR)
(Ministry of Health and Family Welfare, Govt. of India)

Social Marketing Intervention for adverse child-family relationships, Alcohol-usage and Sexual-Health issues attributable to Social-Media-Usage among Adolescents: A Multi-City Cluster Randomized Controlled Trial in North-India.

Social Media and Sexual Health: Awareness, Perception, and Practice Among Adolescents

Section A: Awareness (Knowledge)

No.	Statement	Strongly Disagree	Disagree	Neutral	Agree	Strongly Agree
1.	I am aware that videos or messages on social media can influence a person's sexual health decisions and behaviours. मुझे पता है कि सोशल मीडिया पर वीडियो या संदेश किसी व्यक्ति के यौन स्वास्थ्य निर्णयों और व्यवहारों को प्रभावित कर सकते हैं।					
2.	I am aware that sharing private or intimate photos/messages on social media can lead to serious risks or consequences. मुझे पता है कि सोशल मीडिया पर निजी या अंतरंग तस्वीरें/संदेश साझा करने से गंभीर जोखिम या परिणाम हो सकते हैं।					
3.	I recognise that not all sexual health information shared on social media or by influencers is accurate or trustworthy. मैं मानता हूं कि सोशल मीडिया पर या प्रभावशाली लोगों द्वारा साझा की गई सभी यौन स्वास्थ्य जानकारी सटीक या भरोसेमंद नहीं है।					
4.	I know how to identify reliable or verified social media sources for correct sexual health information. मैं मानता हूं कि सोशल मीडिया पर या प्रभावशाली लोगों द्वारा साझा की गई सभी यौन स्वास्थ्य जानकारी सटीक या भरोसेमंद नहीं है।					
5.	I am aware that interactions on social media can sometimes make individuals vulnerable to sexual manipulation or exploitation. मुझे पता है कि सोशल मीडिया पर बातचीत कभी-कभी व्यक्तियों को यौन हेरफेर या शोषण के प्रति संवेदनशील बना सकती है।					

Section B: Perception (Attitudes and Beliefs)

No.	Statement	Strongly Disagree	Disagree	Neutral	Agree	Strongly Agree
1.	I believe parents and teachers should have open conversations with students about the sexual content they come across on social media. मेरा मानना है कि माता-पिता और शिक्षकों को सोशल मीडिया पर आने वाली यौन सामग्री के बारे में छात्रों के साथ खुली बातचीत करनी चाहिए।					

Funded By
Indian Council of Medical Research (ICMR)
(Ministry of Health and Family Welfare, Govt. of India)

2.	I think young people should check the accuracy of any sexual health information they find on social media before believing or sharing it.<br>मुझे लगता है कि युवाओं को सोशल मीडिया पर मिलने वाली किसी भी यौन स्वास्थ्य जानकारी पर विश्वास करने या साझा करने से पहले उसकी सटीकता की जांच करनी चाहिए।					
3.	I feel that people my age often experience pressure to imitate their sexual health behaviours based on what they see on social media.<br>मुझे लगता है कि मेरी उम्र के लोग अक्सर सोशल मीडिया पर जो देखते हैं, उसके आधार पर अपने यौन स्वास्थ्य व्यवहार की नकल करने के दबाव का अनुभव करते हैं।					
4.	I believe that liking or commenting on sexually suggestive posts may create a negative impression about a person.<br>मेरा मानना है कि यौन विचारोत्तेजक पोस्ट को पसंद करने या टिप्पणी करने से किसी व्यक्ति के बारे में नकारात्मक प्रभाव पड़ सकता है।					
5.	I think social media platforms should enforce stricter rules to limit adolescents' exposure to sexual content.<br>मुझे लगता है कि सोशल मीडिया प्लेटफॉर्म को यौन सामग्री के लिए किशोरों के जोखिम को सीमित करने के लिए सख्त नियमों को लागू करना चाहिए।					

Section C: Sexual Health Practice (Behaviour and Exposure)

No.	Statement	Never	Rarely	Sometimes	Often	Always
1.	I have encountered sexual or sexually suggestive content while using social media.<br>मैंने सोशल मीडिया का उपयोग करते समय यौन या यौन विचारोत्तेजक सामग्री का सामना किया है।					
2.	I continue watching or scrolling through sexual or suggestive content even if it appears unintentionally.<br>मैं यौन या विचारोत्तेजक सामग्री को देखना या स्क्रॉल करना जारी रखता हूं, भले ही यह अनजाने में दिखाई दे।					

Funded By
Indian Council of Medical Research (ICMR)
(Ministry of Health and Family Welfare, Govt. of India)

3.	I have received or been sent private, inappropriate, or sexually explicit messages on social media.<br>मुझे सोशल मीडिया पर निजी, अनुचित, या यौन स्पष्ट संदेश प्राप्त हुए हैं या भेजे गए हैं।					
4.	I have liked, shared, or forwarded posts on social media that contain sexual or suggestive material.<br>मैंने सोशल मीडिया पर उन पोस्ट को लाइक, शेयर या फॉरवर्ड किया है जिनमें यौन या विचारोत्तेजक सामग्री होती है।					
5.	I adjust my privacy settings or block accounts when sexual content on social media makes me feel uncomfortable.<br>मैं अपनी गोपनीयता सेटिंग्स को समायोजित करता हूं या खातों को ब्लॉक करता हूं जब सोशल मीडिया पर यौन सामग्री मुझे असहज महसूस कराती है।					

Section D: Relationships and Interpersonal Experiences

The following questions are about your personal experiences with friendships and relationships. There are no right or wrong answers. Please respond honestly based on your own experience.

No.	Statement	Never	Rarely	Sometimes	Often	Always
1.	I have come across romantic or relationship-related content on social media that has influenced how I think about relationships.<br>मैंने सोशल मीडिया पर रोमांटिक या रिश्ते से संबंधित सामग्री देखी है जिसने रिश्तों के बारे में मेरे सोचने के तरीके को प्रभावित किया है।					
2.	I feel pressure from social media to be in a romantic relationship or to behave in certain ways within a relationship.<br>मैं सोशल मीडिया से रोमांटिक रिश्ते में रहने या रिश्ते के भीतर कुछ खास तरीकों से व्यवहार करने का दबाव महसूस करता हूं।					
3.	I feel pressure from social media to behave in certain ways within a romantic relationship.<br>मैं एक रोमांटिक रिश्ते के भीतर कुछ तरीकों से व्यवहार करने के लिए सोशल मीडिया से दबाव महसूस करता हूं।					
4.	Content I have seen on social media has shaped my understanding of what physical intimacy in a relationship looks like or should look like.<br>सोशल मीडिया पर मैंने जो सामग्री देखी है, उसने मेरी समझ को आकार दिया है कि रिश्ते में शारीरिक अंतरंगता कैसी दिखती है या कैसी दिखनी चाहिए।					

Funded By
Indian Council of Medical Research (ICMR)
(Ministry of Health and Family Welfare, Govt. of India)

5. Which of the following best describes your personal experience with close or romantic relationships?
   निम्नलिखित में से कौन सा घनिष्ठ या रोमांटिक रिश्तों के साथ आपके व्यक्तिगत अनुभव का सबसे अच्छा वर्णन करता है?
   - I have not been in any close or romantic relationship so far
     मैं अब तक किसी भी करीबी या रोमांटिक रिश्ते में नहीं रहा हूं
   - I have had a close friendship that felt romantic but did not involve any physical contact
     मेरी एक करीबी दोस्ती रही है जो रोमांटिक महसूस करती थी लेकिन इसमें कोई शारीरिक संपर्क शामिल नहीं था
   - I have been in a romantic relationship that involved some form of physical closeness without being sexually intimate
     मैं एक रोमांटिक रिश्ते में रहा हूं जिसमें यौन अंतरंग होने के बिना शारीरिक निकटता का कुछ रूप शामिल है
   - I have been in a relationship that involved sexual intimacy
     मैं एक ऐसे रिश्ते में रहा हूं जिसमें यौन अंतरंगता शामिल है
   - I prefer not to answer
     मैं जवाब नहीं देना पसंद करता हूं

Funded By
Indian Council of Medical Research (ICMR)
(Ministry of Health and Family Welfare, Govt. of India)
