# Supplementary material for: Social marketing intervention for social media-related adolescent risk behaviours in North India: A multi-city cluster RCT protocol
Source: PLoS One. 2026 Aug 7;21(8):e0354663. doi: 10.1371/journal.pone.0354663 (PMC13450755; doi:10.1371/journal.pone.0354663)
Supplement: Supplementary file 4 [file pone.0354663.s004.pdf]

Supplementary File -Appendix C- FGD and Interview guides

Purpose and use of this document

This document contains the qualitative data collection instruments developed for Phase 1 (Pre-intervention Formative Research) of the ICMR-funded Multi-City Cluster RCT on Social Marketing Intervention for Social Media-Related Adolescent Risk Behaviours.

Two types of instruments are included:

- Focus Group Discussion (FGD) Guides- two versions, one each for Students, Parents, and Teachers.
- Qualitative Interview Guides- two versions, one each for Subject Experts, Stakeholders (students, parents, teachers), and Key Informants.

General instructions for facilitator

- All FGDs and interviews must be conducted in the preferred language of participants (Hindi or English).
- Written informed consent/assent must be obtained before beginning any session.
- Sessions must be audio-recorded (with prior consent).
- FGDs should be conducted separately for boys and girls in the student group to allow candid responses.
- The facilitator should not express personal opinions or lead participants toward particular answers.
- Sensitive topics (sexual health, alcohol use) should be approached with cultural sensitivity and non-judgmental language.
- Field notes should be maintained by a co-facilitator throughout each session.

Note to the facilitator: The questions listed under each section are guiding questions. Follow the natural flow of conversation. Use probes to elicit depth and detail. Not all probes need to be used in every session; use professional judgement based on participant response.

PART I: Focus Group Discussion

Minimum 6 FGDs in total (2 per study area: Chandigarh, Mohali, Panchkula). Each FGD includes 8–12 participants. Separate FGDs will be conducted for each stakeholder group.

FGD Guide 1: Adolescents/students

**Target Group**	School-going adolescents aged 14–17 years (Grades 9–12)
**Group Size**	8–12 participants per FGD; conducted separately for boys and girls
**Duration**	60–90 minutes
**Setting**	A private, comfortable room within the school premises
**Facilitator**	Trained researcher; co-facilitator for field notes and recording
**Language**	Hindi / English (as preferred by participants)
**Recording**	Audio-recorded with prior written assent and parental consent

Introductory Script

"Hello, everyone. Thank you for joining us today. My name is [Facilitator Name], and I am a researcher from PGIMER, Chandigarh. We are doing a study to understand how young people like you use social media and how it affects your daily life, your relationships with your family, and your health. There are no right or wrong answers; we want to hear your honest opinions and experiences. Everything you share here will be kept confidential and will only be used for research purposes. Your name will never be used in any report. This discussion will be audio-recorded, but only the research team will have access to the recordings. Do you have any questions before we begin? May we start?"

Section 1: Warm-Up and Social Media Use Patterns

1. Let's start with something you all know well, social media. Which social media platforms do you use most these days?

   Probe: Which one do you spend the most time on? About how many hours a day?

   Probe: Do you use it more on weekdays or weekends?

2. When and where do you usually use social media? (e.g., at home, school, at night, during meals)

   Probe: Do you use it after your parents/family go to sleep?

   Probe: Do you use social media during school hours?

3. Who in your home uses social media? Do your parents or siblings also use it?

   Probe: Do your parents know what you do on social media?

Probe: Are there any rules at home about how much time you can spend on your phone?

Section 2: Perceived Impact on Family Relationships

4. Has using social media ever caused any problems or disagreements at home? Can you share an example?

   Probe: Have you ever felt that social media takes time away from spending time with your family?

   Probe: Has it ever led to arguments with parents or siblings?

5. Do you feel that you and your family communicate differently because of social media?

   Probe: Do you talk less face-to-face now? Or do you communicate more through messages/calls?

   Probe: Has social media ever helped you feel closer to a family member?

6. Do you think your parents understand how you use social media? Why or why not?

   Probe: Have your parents ever expressed concern about your social media use?

   Probe: How did that make you feel?

7. In your opinion, what are the biggest ways social media changes relationships between young people and their parents or family?

Section 3: Social Media and Alcohol Use

FACILITATOR: Approach this section casually and non-judgmentally. Do not use the word 'alcohol' initially; let participants introduce it. Ensure confidentiality is re-emphasized before this section.

8. What kind of content do you usually see on social media, videos, reels, posts? What's popular among young people your age?

   Probe: Do you ever see content related to parties, drinking, or celebrations? What do you think about that content?

9. Have you ever seen friends or people your age posting about drinking or using alcohol on social media?

   Probe: How does seeing that kind of content make you feel?

   Probe: Does it make you curious? Does it make you want to try it?

10. In your experience, does social media make alcohol seem cool or normal among young people?

    Probe: Can you give an example of content you have seen that gives that impression?

11. Do you think young people your age drink because of what they see on social media? Or are there other reasons?

Probe: What do you think would help young people make better choices about alcohol?

Section 4: Social Media and Sexual Health

FACILITATOR: This is a sensitive section. Reassure participants of confidentiality. Use neutral, non-judgmental language. Conduct this section with single-gender groups only.

12. What kinds of things do you see on social media about relationships and romance?

    Probe: Do you think what you see on social media gives a realistic picture of relationships?

    Probe: Has social media ever influenced the way you think about relationships?

13. Do you feel that there is pressure on young people to behave in certain ways in relationships because of what they see on social media?

    Probe: Have you or your friends ever felt pressured to do something in a relationship because of what you saw online?

14. Where do young people your age usually get information about sexual health — school, parents, internet, social media?

    Probe: Do you think the information available on social media about sexual health is accurate and trustworthy?

    Probe: What kind of information would you actually want to have?

15. Have you ever encountered uncomfortable or inappropriate content on social media? How did you handle it?

    Probe: Did you talk to anyone about it? A friend, parent, or teacher?

16. What do you think schools or parents should do to help young people handle sexual content on social media better?

FGD Guide 2: Parents/Guardians/Teachers

**Target Group**	Parents or legal guardians of adolescents (aged 14–17) enrolled in study schools
**Group Size**	8–12 participants per FGD
**Duration**	60–90 minutes
**Setting**	School meeting room or community hall — private and comfortable
**Facilitator**	Trained researcher; co-facilitator for field notes and recording
**Language**	Hindi / English (as preferred by participants)
**Recording**	Audio-recorded with prior written informed consent

Introductory Script

"Hello. Thank you for giving us your valuable time today. We are researchers from PGIMER, Chandigarh, conducting a study on how social media use among school-going adolescents affects their health and their relationships with their families. As parents, your perspective is extremely important to us. There are no right or wrong answers. Everything shared today is confidential and will only be used for research purposes. We will not share anything you say with the school, your children, or anyone else. The session will be audio-recorded. Do you have any questions before we begin?"

Section 1: Awareness of Adolescent Social Media Use

1. Tell me about how your child uses social media. How much time do they spend on it?

   Probe: Do you know which platforms they use?

   Probe: Do you know what kind of content they watch or share?

2. Do you have any rules or limits at home about how your child uses social media or their phone?

   Probe: How easy or difficult has it been to enforce these rules?

   Probe: Has your child ever tried to hide their social media use from you?

3. How comfortable do you feel using social media yourself? Do you feel you understand it well enough to guide your child?

   Probe: Have you ever looked at your child's social media account?

   Probe: What do you feel you need to know more about to help your child stay safe online?

Section 2: Impact on Family Relationships

4. Has social media use changed the way your family interacts with each other at home?

Probe: Has it reduced the time spent together as a family?

Probe: Has it caused any conflicts or arguments?

5. Do you feel you have a good understanding of what your child is going through because of social media?

   Probe: Do you feel able to talk to your child openly about their social media use?

   Probe: What makes these conversations easy or difficult?

6. Have you noticed any changes in your child's behaviour, mood, or sleep that you think might be linked to their social media use?

   Probe: Were you able to address these changes? How?

7. What do you think are the biggest threats that social media poses to the parent-child relationship?

Section 3: Social Media and Alcohol Use

8. Are you aware that a lot of alcohol-related content is available on social media platforms that your child uses?

   Probe: Has your child ever talked to you about seeing such content?

   Probe: Have you ever come across such content while looking at your child's phone?

9. Do you think social media exposure to alcohol-related content influences young people's behaviour?

   Probe: Do you think your child is at risk?

   Probe: How have you discussed the issue of alcohol with your child?

10. What would make you more confident in talking to your child about alcohol and the influence of social media?

Section 4: Social Media and Sexual Health

Facilitator Note: Approach with sensitivity. Use respectful, non-clinical language.

11. Are you aware that adolescents are frequently exposed to sexual content on social media?

    Probe: Has your child ever come to you with any questions or concerns related to this?

    Probe: How comfortable are you talking to your child about sexual health topics?

12. How do you think exposure to sexual content on social media affects young people's attitudes and behaviour?

    Probe: Do you think schools should play a role in addressing this?

13. What kind of guidance or support would you want schools or health professionals to provide to help parents in this area?

Section 5: Views on a Social Marketing Intervention

14. If a programme was developed to help adolescents use social media more safely and responsibly, what would you want it to include?

    Probe: Would you want parents to be involved? In what way?

    Probe: How should it be delivered — in school, at home, through social media itself?

15. What do you think would be the biggest barriers to young people changing their social media habits?

    Probe: What would motivate them to change?

16. What would make you, as a parent, more involved and supportive of such a programme?

Closing

17. Is there anything else you would like to share about this topic that we have not covered?

FACILITATOR: Thank participants sincerely. Assure confidentiality once more. Provide school/project contact details if they have further concerns.

PART II: Qualitative Interview Guides

30 qualitative interviews in total: 10 per study area (Chandigarh, Mohali, Panchkula). Interviews will be conducted with subject experts and stakeholders in a proportionate distribution. Each interview is individual, in-depth, and semi-structured.

Interview Guideline 1: Subject experts

(Mental Health Professionals / Social Scientists / Reproductive Health Experts / Public Health Researchers)

**Target Group**	Experts in mental health, social science, reproductive health, and public health (3–4 per study area)
**Duration**	45–60 minutes
**Mode**	In-person or video call, as feasible
**Setting**	An expert's workplace or a mutually agreed private setting
**Recording**	Audio-recorded with prior written informed consent
**Sampling**	Purposive sampling; experts with direct professional experience with adolescent populations

Introductory Script

"Thank you for agreeing to participate in this interview. I am a researcher from PGIMER, Chandigarh, working on an ICMR-funded study on social media use and adolescent health in North India. We are in the formative phase of developing a social marketing intervention for school-going adolescents. Your expert perspective will be invaluable in shaping the intervention. The interview will take approximately 45–60 minutes. With your permission, it will be audio-recorded. Everything you share will be kept confidential and used solely for research purposes. May we begin?"

Section 1: Understanding the Problem

1. From your professional experience, what are the most significant ways in which excessive social media use is affecting adolescent health and well-being in India?

   Probe: Which outcomes concern you the most: mental health, substance use, sexual behaviour, family dynamics?

   Probe: Are there patterns unique to the North Indian or urban/peri-urban context?

2. How do you assess the current evidence based on the relationship between social media use and adolescent risk behaviours in LMIC settings like India?

   Probe: What are the key gaps in this evidence?

Probe: What methodological approaches do you think work best for studying this population?

3. In your view, how does social media specifically influence adolescent alcohol use and sexual behaviour? What are the pathways?

   Probe: Is the influence primarily through content exposure, peer norming, or something else?

   Probe: How does it differ between boys and girls?

Section 2: Family and Relational Dimensions

4. How do you assess the impact of adolescent social media use on child-family relationships in Indian households?

   Probe: What family-level factors moderate or exacerbate this impact?

   Probe: How do parental digital literacy and supervision practices play a role?

5. What specific aspects of the parent-child relationship should a school-based intervention aim to strengthen?

   Probe: Are there evidence-based strategies from your field that could be adapted?

Section 3: Intervention Design

6. We plan to use the COM-B model to design our social marketing intervention. How do you think capability, opportunity, and motivation should each be addressed in an adolescent-focused intervention of this kind?

   Probe: Which component do you think is most deficient in the target population — capability, opportunity, or motivation?

   Probe: How would you prioritise the three components?

7. What behaviour change techniques (BCTs) do you consider most effective for adolescents in the context of social media use and its associated risk behaviours?

   Probe: In your experience, what has worked and what has not worked in previous school-based interventions?

8. How should a social marketing intervention in schools be structured to ensure cultural appropriateness in the North Indian context?

   Probe: What cultural values or norms should the intervention acknowledge or work with?

   Probe: What topics or approaches should be handled with particular care?

Section 4: Measurement and Evaluation

9. What key outcomes should be measured to evaluate the effectiveness of this intervention?

   Probe: What tools or scales would you recommend?

   Probe: Are there any validated Hindi instruments you would suggest?

10. What do you consider the most important process indicators to track during intervention delivery?

11. What are the key methodological challenges you foresee in conducting a cluster RCT in school settings in this region?

    Probe: How would you address potential contamination between intervention and control schools?

Section 5: Scale-up and Policy Relevance

12. How do you see this intervention fitting within existing school health programmes such as the Ayushman Bharat School Health and Wellness Programme?

    Probe: *What would be needed to make it scalable and sustainable?*

13. What policy recommendations would you make to address adolescent social media-related health risks in India?

Closing

14. Is there any other insight, recommendation, or resource you would like to share that we have not covered?

Facilitator: Thank the expert. Ask if they are willing to be contacted for follow-up clarifications. Provide project contact details.

Interview Guide 2: Stakeholders

(Students, Parents, and Teachers — individual in-depth interviews)

**Target Group**	Students (14–17 yrs), Parents, and Teachers from study schools (proportionate distribution)
**Duration**	30–45 minutes per interview
**Mode**	In-person, in a private setting within the school or home
**Recording**	Audio-recorded with prior written assent/consent
**Sampling**	Purposive sampling to ensure diversity in gender, class, school type, and city

Introductory Script

"Hello, thank you for agreeing to speak with me today. I am a researcher from PGIMER, Chandigarh. I would like to understand your personal experiences and opinions about social media and how it affects your life and the lives of young people around you. Your responses will be kept completely confidential. Nothing you say will be shared with your school, your parents/child, or anyone else. You can stop the interview at any time if you feel uncomfortable. May I record our conversation? Let us begin."

Section A: For Students

Social Media Use — Personal Experience

1. Can you tell me a bit about how you use social media in your daily life?

   Probe: How many hours a day would you say you spend on it?

   Probe: What do you use it for, entertainment, connecting with friends, or information?

2. Have you ever felt like social media was taking up too much of your time or affecting your life negatively?

   Probe: Did you try to do anything about it? Did it work?

Family Relationships

3. How do you think social media has changed the way you relate to your family?

   Probe: Has it caused any conflicts at home?

   Probe: Do you feel your parents understand your world on social media?

4. Is there something you wish your parents understood better about your social media use?

Alcohol and Social Media

5. Do you see alcohol-related content on social media? How does it make you feel?

   Probe: Do you think it influences how young people think about or use alcohol?

Sexual Health and Social Media

FACILITATOR: Ensure single-gender setting. Reassure confidentiality before this section

6. Do you ever come across content on social media about relationships or sexual health?

   Probe: Do you think this content is helpful or harmful? Why?

   Probe: Where would you prefer to get reliable information about sexual health?

Views on the Intervention

7. If your school offered a programme to help students use social media more safely, what would you want it to look like?

   Probe: What topics would you want covered?

   Probe: How should it be delivered — classroom sessions, social media, peer-led?

Section B: For Parents

Awareness and Supervision

8. Tell me how you manage your child's social media use at home.

   Probe: Do you feel you are aware of what your child does on social media?

   Probe: What challenges do you face?

9. Have there been any situations at home related to your child's social media use that concerned you?

   Probe: How did you handle it?

Family Communication

10. How has social media changed communication in your family?

    Probe: Do you feel able to talk openly with your child about what they see and do on social media?

    Probe: What topics do you find most difficult to discuss?

Alcohol and Sexual Health

11. Are you concerned about your child being exposed to alcohol-related or sexual content on social media?

Probe: How have you addressed this with your child?

Probe: What support do you feel you need?

Views on the Intervention

12. What would a good school-based programme look like to you as a parent?

    Probe: Would you want to be involved? How?

    Probe: What would make you confident that such a programme is appropriate for your child?

Section C: For teachers

Classroom and School Context

13. From your daily interactions with students, what do you think is the biggest challenge that social media poses for adolescents today?

    Probe: How does it manifest in the classroom?

    Probe: What do students themselves say about it?

14. Have you ever had to address a situation in school that was linked to a student's social media use?

    Probe: What happened? How did you handle it?

Alcohol and Sexual Health

15. Do you feel the school provides adequate guidance to students on alcohol use and sexual health in the context of social media?

    Probe: What is currently being done? What is missing?

Teacher Role in the Intervention

16. This study envisions teachers as key facilitators of the intervention. How do you feel about taking on that role?

    Probe: What training would you need?

    Probe: What would make you feel confident and comfortable facilitating these sessions?

17. What do you think is the single most important change a school-based intervention should aim to bring about in adolescents?

Closing

18. Is there anything else you would like to add or share?

FACILITATOR: Thank the participant. Remind them of confidentiality. Provide the school counsellor/project contact if they have any concerns after the interview.

Interview Guide 3: Key Informants

(School Principals, District Education Officers, Child Rights Officials, NGO Representatives)

**Target Group**	School principals, education administrators, child rights officials, NGO workers with adolescent focus
**Duration**	45–60 minutes
**Mode**	In-person or telephonic/video call
**Recording**	Audio-recorded with prior written informed consent
**Sampling**	Purposive; individuals with decision-making or implementation authority in school health or adolescent welfare

Introductory Script

"Thank you for taking the time to speak with us. I am a researcher from PGIMER, Chandigarh, working on an ICMR-funded study on the impact of social media on adolescent health in North India. We are developing a school-based intervention and want to understand the broader institutional and community context. Your perspective as a [principal/official/NGO leader] is extremely important. This interview is confidential and will be used only for research purposes. May I record our conversation? Let us begin."

Section 1: Institutional Context and Current Challenges

1. From your position, how significant do you consider the issue of excessive social media use among school-going adolescents in this region?

   Probe: Is this something that comes to your attention regularly?

   Probe: What are the most common concerns you hear about?

2. What systems or policies currently exist in your institution/department to address digital safety, alcohol use, or sexual health among adolescents?

   Probe: How effective do you think these are?

   Probe: What gaps exist?

3. What are the most significant structural or systemic barriers to addressing these issues effectively in schools in this area?

   Probe: Is it funding? Capacity? Parental resistance? Curriculum constraints?

Section 2: Community and Cultural Context

4. Are there particular cultural norms or community attitudes in Chandigarh/Mohali/Panchkula that affect how adolescent health issues, especially around alcohol and sexual health, are discussed and addressed?

   Probe: Are there differences between government and private school contexts?

   Probe: Are there differences between communities that the intervention should be sensitive to?

5. How do you think parents in this region generally respond to school-initiated health programmes?

   Probe: What has worked in the past to get parental buy-in?

6. Are there any existing community resources or networks (NGOs, health centres, community leaders) that could be leveraged for this intervention?

Section 3: Views on Intervention Design and Implementation

7. We are planning a three-month social marketing intervention delivered through schools, involving teachers as facilitators and using IEC materials alongside social media channels. What do you think about this approach?

   Probe: What are its strengths and weaknesses from an implementation standpoint?

   Probe: What risks or challenges do you anticipate?

8. How should schools be engaged and motivated to participate actively in such an intervention?

   Probe: What kind of institutional support or incentives would help?

9. What would make this intervention sustainable beyond the research period?

   Probe: Could it be integrated into existing programmes like Ayushman Bharat SHWP?

   Probe: What would need to happen at the policy level?

Section 4: Data and Ethics

10. From your experience, what are the key ethical considerations in conducting health research with adolescents in school settings in this region?

    Probe: Specifically, around sensitive topics like sexual health and alcohol?

    Probe: What is the best way to ensure parental informed consent while also respecting adolescent confidentiality?

11. Are there any data protection or community-level concerns we should be aware of as we conduct this study?

Closing

12. Is there any advice, concern, or recommendation you would like to offer before we close?

FACILITATOR: Thank the key informant sincerely. Explore the possibility of an ongoing advisory role during intervention implementation if appropriate. Provide project contact details.
