# Supplementary material for: Social marketing intervention for social media-related adolescent risk behaviours in North India: A multi-city cluster RCT protocol
Source: PLoS One. 2026 Aug 7;21(8):e0354663. doi: 10.1371/journal.pone.0354663 (PMC13450755; doi:10.1371/journal.pone.0354663)
Supplement: Supplementary file 5 [file pone.0354663.s005.pdf]

Supplementary File (Appendix D)

Tentative Components of the Proposed Social Marketing Intervention

Social marketing is highly effective in encouraging people to engage in proper health behaviours. Various studies reported successful campaigns that used this approach all around the world. Social marketing is the application of marketing techniques and strategies to the analysis, planning, execution, and evaluation of programs designed to influence the voluntary behaviour of target audiences to improve their health outcomes, personal welfare, and that of their society (Nejad et al., 2021). Based on the literature, we have identified tentative domains of the proposed social marketing intervention as shown in **Figure 1.** It primarily involves the use of behaviour modification techniques explained below:

**Proposed Components Based on preliminary literature review**		**Description of the Proposed Intervention**
**Month 1**<br>Impactful group counselling and awareness sessions<br>**30-45 minutes monthly session**<br>**+**<br>**Relevant IEC material**	**Social Media Usage**	• Group counselling will be given about Social Interaction and social platforms.<br>• Awareness classes about Mental Health Impact<br>• Information Booklets & Information about accessing govt deaddiction centres & rehab centres will be given for Psychosocial Impact .<br>• social marketing using social media (sharing infographics, flyers and pamphlets etc).
	**Alcohol Usage**	• Group counselling, Awareness classes, Information Booklets, social marketing using social media (sharing infographics, flyers and pamphlets etc) for Peer Influence, Binge drinking, Alcohol abuse, Risk-Taking Behavior & Information about accessing govt deaddiction centres & rehab centres.
	**Sexual Health Issues**	• Separate gender based counselling<br>• Awareness classes, social marketing using social media (sharing infographics, flyers and pamphlets etc) & Information Booklets about Sexual Education, Peer Influence and Media Impact & Information about accessing govt delivery of reproductive services.
	**Adverse family relationship issues**	• Family-child sessions for bringing intimacy & direct communication between child & parents, Conflicts management sessions regarding social media usage and Miscommunication, & Parental Understanding of social media..
**Month 2**<br>In addition to activity described in first month, social marketing for behavioral change is planned		Leverage the power of social media platforms<br>**+**<br>Infographics, flyers, and pamphlets will be created and shared across these social media platforms
**Month 3**<br>In addition to activity described in first and second month,<br>Dynamic approach by Combining social marketing campaigns and extracurricular activities is planned, coinciding with the parent-teacher meeting at the school.		**+**<br>Dedicated radio slots, Facebook pages, Whatsapp group messages, newspaper advertisement to market the planned campaign<br>**+**<br>Extra Curricular activities involving child-parent direct interaction like quiz competition, slogan writing, 2 minutes speak up

Figure 1- Tentative Components of the proposed social marketing intervention
