# Supplementary material for: Social marketing intervention for social media-related adolescent risk behaviours in North India: A multi-city cluster RCT protocol
Source: PLoS One. 2026 Aug 7;21(8):e0354663. doi: 10.1371/journal.pone.0354663 (PMC13450755; doi:10.1371/journal.pone.0354663)
Supplement: Supplementary file 6 [file pone.0354663.s006.pdf]

Supplementary Material Appendix E: GEE model equations

Generalised Estimating Equations

Social Media use $= \beta_0 + \beta_1 T_1 + \beta_2 T_2 + \beta_3 T_3 + \beta_4 T_4 + \beta_5\, Intervention\ Group + \beta_6 (T_1 * Intervention\ Group) + \beta_7 (T_2 * Intervention\ Group) + \beta_8 (T_3 * Intervention\ Group) + \beta_9 (T_4 * Intervention\ Group) + e$

Adverse family-child relationship $= \alpha_0 + \alpha_1 T_1 + \alpha_2 T_2 + \alpha_3 T_3 + \alpha_4 T_4 + \alpha_5 Intervention\ Group + \alpha_6 (T_1 * Intervention\ Group) + \alpha_7 (T_2 * Intervention\ Group) + \alpha_8 (T_3 * Intervention\ Group) + \alpha_9 (T_4 * Intervention\ Group) + e$

Alcohol usage $= \gamma_0 + \gamma_1 T_1 + \gamma_2 T_2 + \gamma_3 T_3 + \gamma_4 T_4 + \gamma_5 Intervention\ Group + \gamma_6 (T_1 * Intervention\ Group) + \gamma_7 (T_2 * Intervention\ Group) + \gamma_8 (T_3 * Intervention\ Group) + \gamma_9 (T_4 * Intervention\ Group) + e$

Sexual Health Issues $= \zeta_0 + \zeta_1 T_1 + \zeta_2 T_2 + \zeta_3 T_3 + \zeta_4 T_4 + \zeta_5 Intervention\ Group + \zeta_6 (T_1 * Intervention\ Group) + \zeta_7 (T_2 * Intervention\ Group) + \zeta_8 (T_3 * Intervention\ Group) + \zeta_9 (T_4 * Intervention\ Group) + e$

Adjusted GEE model → ***Outcome variable*** $= \delta_0 + \delta_1 T_1 + \delta_2 T_2 + \delta_3 T_3 + \delta_4 T_4 + \delta_5 Intervention\ Group + \delta_6 (T_1 * Intervention\ Group) + \delta_7 (T_2 * Intervention\ Group) + \delta_8 (T_3 * Intervention\ Group) + \delta_9 (T_4 * Intervention\ Group) + \sum_{j=1}^{n} \beta_j X_j + e$

Where:

T_0 = Baseline assessment (Reference Category)

T_1 = First Follow-up (Immediately post-intervention

T_2 =Passive Follow-up 1

T_3 = Passive Follow-up 2

T_4= Passive Follow-up 3

Control Group = Reference Group

$\beta_0, \alpha, \gamma, \zeta, \delta$ = Intercepts for outcomes

e = error term

X_j= covariates included in the adjusted model
